# Early Enteral vs Oral Postoperative Nutrition After Pancreatoduodenectomy: The NUTRIWHI Randomized Clinical Trial

**DOI:** 10.1001/jamasurg.2026.1048

**Published:** 2026-04-22

**Authors:** Gaëtan-Romain Joliat, David Martin, Ismail Labgaa, Emilie Uldry, Emmanuel Melloul, Nermin Halkic, Alessandra Cristaudi, Pietro Majno-Hurst, Gioia Pozza, David Fuks, Ugo Marchese, Nicolas Demartines, Markus Schäfer

**Affiliations:** 1 Department of Visceral Surgery, Lausanne University Hospital CHUV, University of Lausanne (UNIL), Lausanne, Switzerland; 2 Graduate School of Health Sciences, University of Bern, Bern, Switzerland; 3 Department of Surgery, Regional Hospital of Lugano, Lugano, Switzerland; 4 Department of Digestive, Pancreatic, Hepatobiliary and Endocrine Surgery, Cochin Hospital, Paris, France

## Abstract

**Importance:**

The optimal type of nutritional support after pancreatoduodenectomy remains controversial.

**Objective:**

To compare postoperative early enteral nutrition (EEN) with oral nutrition (ON) in patients nutritionally at risk in terms of postoperative morbidity.

**Design, Setting, and Participants:**

This study was a parallel, open-label, superiority randomized clinical trial that took place at 3 tertiary centers in Switzerland and France. Patients at increased nutritional risk (nutritional risk screening score ≥3) were randomized 1:1 into the EEN or ON groups. Patients in the EEN group received enteral nutrition immediately after surgery via a nasojejunal tube placed intraoperatively and were allowed to have oral food based on the same protocol as the other group. Patients in the ON group were not allowed to have enteral nutrition during hospitalization. In both groups, parenteral nutrition need was standardized. These data were analyzed from February 2025 to April 2025.

**Intervention:**

EEN via nasojejunal tube vs ON.

**Main Outcomes and Measures:**

Primary outcome was complications at 90 days postoperatively, defined by the mean comprehensive complication index. Secondary end points were morbidity rates (overall, minor, and major complications), mortality, delayed gastric emptying, pancreatic fistula, postoperative hemorrhage, surgical site infections, infectious complications, pulmonary complications, length of stay, and 90-day readmission.

**Results:**

Patients were enrolled from December 15, 2021, through October 8, 2024. A total of 144 patients were included and 142 patients were randomized. After randomization, 24 patients dropped out (17%), leaving 118 patients for analysis (59 in the EEN group and 59 in the ON group). Patients in the EEN group had a lower mean 90-day comprehensive complication index compared with ON patients (mean [SD], 25.5 [21.1] vs 35.8 [25.2]; mean difference 10.3; 95% CI 1.8-18.8; *P* = .02). At 90 days, overall morbidity rates were 45 of 59 and 51 of 59 in the EEN and ON groups (risk ratio [RR], 1.13; 95% CI, 0.9-1.9; *P* = .18). No difference was found between both groups regarding specific complications (delayed gastric emptying, pancreatic fistula, postoperative hemorrhage, and surgical site infection). In the EEN group, nasojejunal tube was replaced in 14 patients due to involuntary removal (adverse event).

**Conclusions and Relevance:**

In this randomized clinical trial, in patients with a nutritional risk screening score of 3 or more, EEN after pancreatoduodenectomy permitted to decrease the burden and incidence of postoperative complications compared with ON.

**Trial Registration:**

ClinicalTrials.gov Identifier: NCT05042882

## Key Points

**Question:**

Does early supplemental enteral nutrition after pancreatoduodenectomy improve postoperative morbidity compared with oral nutrition?

**Findings:**

In this randomized clinical trial that included 144 patients, patients with early supplemental enteral nutrition had a statistically significant lower mean comprehensive complication index (25.5) than patients with oral nutrition (35.8).

**Meaning:**

In malnourished patients, use of early supplemental enteral nutrition should be routinely considered, as its global impact could be significant considering the high morbidity rates after pancreatoduodenectomy.

## Teaser

This randomized clinical trial compares postoperative early enteral nutrition with oral nutrition in patients nutritionally at risk in terms of postoperative morbidity.

## Introduction

Despite many technical improvements, pancreatoduodenectomy (PD) remains a challenging operation with high morbidity rates (50%-70%).^1,2^ Postoperative complications are clinically relevant as they impair patient’s quality of life and long-term survival, as adjuvant chemotherapy is postponed or dose reductions cannot be avoided.^3^ Hence, early detection and minimizing postoperative complications are at the top of the bucket list of pancreatic outcome research.

The presence of malnutrition is a major risk factor for complications after PD.^4,5,6^ A maintained perioperative nutritional state is paramount to maximizing the healing process and permit optimal recovery after surgery.

Patients scheduled to undergo PD who are at nutritional risk should be treated preoperatively using oral nutritional supplements, enteral nutrition, or even parenteral nutrition (PN).^7,8^ Postoperative nutritional support should provide adequate caloric intake and protein requirements during the recovery period. There is an ongoing debate whether PN or enteral nutrition (EN) should be preferred postoperatively.^9,10,11^ Routine oral nutrition (ON) is currently the recommended option after PD based on the Enhanced Recovery After Surgery (ERAS) guidelines, with artificial nutrition reserved for individual patients according to their nutritional status assessment.^8^ However, few comparative articles and robust data currently evaluate ON vs early EN after PD in malnourished patients. The authors think that as ON is often not sufficient to cover all required protein and caloric needs during the first postoperative days after PD, adjunct of early EN might bring all required nutrients and calories to patients to face the catabolic postoperative phase. This well-balanced protein and caloric intake could allow patients to better face surgical stress with potentially less risk of complication, especially in malnourished or at-risk patients. This current randomized clinical trial (RCT) aimed to assess the impact of early supplemental EN in addition to ON compared with ON alone after PD on the occurrence of postoperative complications.

## Methods

### Study Design

This study was a parallel, open-label, multicentric, superior randomized clinical trial. Three centers participated in the study: Lugano Regional Hospital (Lugano, Switzerland), Cochin Hospital (Paris, France), and Lausanne University Hospital CHUV (Lausanne, Switzerland). This trial was registered on ClinicalTrials.gov (NCT05042882). This study was approved by the referral and main ethical committee (Commission cantonale d'éthique de la recherche sur l'être humain; 2021-00724). This article followed the Consolidated Standards of Reporting Trials (CONSORT) reporting guidelines.^13^

### Patients

All consecutive patients seen in the outpatient department who were scheduled for PD were screened. Patients were eligible if they had a preoperative nutritional risk screening (NRS) score of 3 or more.^12^ Patient age younger than 18 years, preexisting psychological disorders, preoperative EN, and major language barrier were exclusion criteria. All patients had to sign the study consent form. Once included, the patients were randomized within 24 hours before the operation and not informed of their allocation group. Patients with intraoperatively confirmed nonresectable tumors, requiring a suction nasogastric tube (NGT) at the operation end, or who decided to withdraw their consent to participate were excluded and considered dropouts. Patients underwent classic or pylorus-preserving PD, which was left to the individual surgeon’s discretion. The inclusion period was from December 15, 2021, to October 8, 2024. The end of the follow-up (90 days postoperative) of the last included patient was January 13, 2025.

### Randomization and Masking

Randomization was performed in REDCap the day before surgery or on the day of surgery once the patient had signed the informed consent form and was included. Randomization was stratified based on the centers with the same patient number in the interventional (enteral) and oral groups (1:1). Randomization was performed by mixed-variable block sizes of 4, 6, and 8 randomly selected. A randomization table was created by a statistician in REDCap. The patients and surgeons were not blinded to the intervention. Assessment of the complications and calculation of the Comprehensive Complication Index (CCI) for each patient was done without knowing the nutrition group allocation.

### Procedures

Patients were randomized to receive either ON and supplemental EN or ON alone after PD (parallel-group design). Patients included in the supplemental EN group received the same standardized ON as patients in the other group but received additional EN through a nasojejunal tube (NJT) based on a standardized algorithm (see trial protocol in Supplement 1 and eMethods in Supplement 2).^14^ Patients in the EN group had an NJT (8-French Freka polyurethane tube) placed under direct vision intraoperatively (30 cm distal to the gastrojejunal anastomosis).

PN use was similarly standardized in both groups. PN through a central catheter was initiated if the daily caloric intake was less than 50% of caloric requirements on postoperative day 3. PN was continued until the total caloric intake, without the PN, reached more than 50% of daily caloric needs, and until an NGT was no longer in place.

### Outcomes

The primary end point was the mean CCI on postoperative day 90.^15^ The CCI is an overall index that summarizes all complications a patient develops (from 0 = no complication to 100 = death), giving an easily understandable summary of morbidity. It was chosen as primary end point as it reflects the whole burden of complications for a patient giving more information on real morbidity among patients having more than 1 complication and because it is a continuous variable.^16^ The CCI has also been shown to be more sensitive in detecting differences between groups in RCTs than the Clavien-Dindo classification.^17^ Secondary end points included morbidity rates (overall, minor, and major complications), mortality, delayed gastric emptying (DGE), pancreatic fistula, postoperative hemorrhage, surgical site infections (SSI), infectious complications (all infections occurring postoperatively except SSI), pulmonary complications (all postoperative complications affecting the lungs or respiratory system, such as pneumonia, pulmonary embolism, or pleural effusion), length of stay (LOS), and 90-day readmission. Complications were defined and graded based on the Clavien-Dindo classification.^18^ Minor complications were defined as grade 1 to 2, while major complications were defined as grade 3a or higher. Mortality was classified as grade 5. DGE, pancreatic fistula, and postoperative hemorrhage were defined according to the International Study Group for Pancreatic Surgery.^19,20,21^ SSI were classified according to the US Centers for Disease Control and Prevention.^22^ The NRS was calculated based on the definition provided by Kondrup et al.^12^ This score ranges from 0 to 7 and is subdivided into 3 parts (nutritional status, disease severity, and age). Nutritional requirements per day were defined as 30 kcal/kg in patients with body mass index (BMI) (calculated as weight in kilograms divided by height in meters squared) less than 30 and 25 kcal/kg in patients with BMI higher than 30.

All patients followed an ERAS pathway specific to PD.^8^ In case of DGE, a suction NGT was inserted and, if patients were in the EN group, the NJT was maintained if possible.

### Statistical Analysis

Based on the previous literature results, it was hypothesized that EN would reduce a mean (SD) CCI of 35 (20) by 30% in the ON group, with an expected mean (SD) CCI for the EN group of 24.5 (20). In this superiority study, for a power of 80% and a significance level less than or equal to .05 (2-sided α), it was calculated that 57 patients per group were necessary. Assuming a 20% dropout rate, the total sample size calculation was 144 patients (72 patients per group). Patient inclusion was stopped once the target sample size was reached. Categorical data were summarized with number and percentage, while continuous data were summarized with mean and SD (including the primary outcome, CCI) or median and IQR. Comparisons between groups (oral vs enteral) were presented as risk ratios (RRs) with 95% CIs for categorical data and mean differences with 95% CIs for continuous data, and differences were calculated using χ^2^ tests and *t *tests or Mann-Whitney *U* tests depending on the variable normality, respectively.

Analyses were performed in a modified intention-to-treat manner (defined as all randomized patients who underwent PD, each group defined by the allocation of randomization) first and completed by a per-protocol analysis (groups based on the nutrition finally received). Moreover, a sensitivity analysis of the primary outcome was performed without including patients who died during the 90-day postoperative period. Lastly, center-adjusted analyses of the primary end point (CCI) were performed (subgroup analysis and multiple linear regression).

Subgroup analyses for the primary outcome (CCI) were performed on a post hoc basis according to factors potentially influencing the postoperative morbidity: age (≤65 years, >65 years), diabetes (yes/no), BMI (≤25, >25), preoperative biliary drainage (yes/no), and NRS score (3, >3). For these analyses, Bonferroni-adjusted *P* value was used (1%).

Otherwise, a *P* value <.05 was considered significant. All statistical tests were 2-sided. All statistics were performed using SPSS version 29.0 for MacOSX (IBM). More details on the methods can be found in Supplement 1 and Supplement 2.^14^

## Results

**Figure 1.**
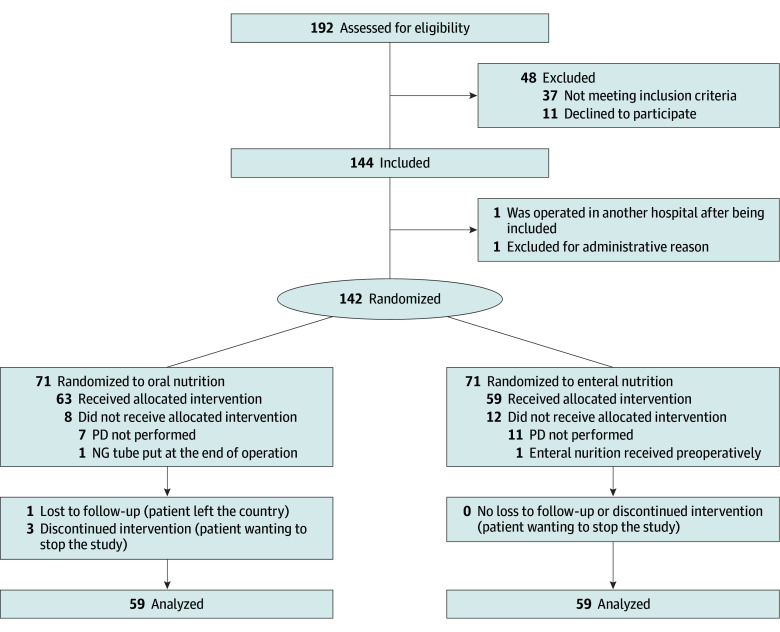
Study Flowchart NG indicates nasogastric; PD, pancreatoduodenectomy.

Patients were recruited from December 15, 2021, through October 8, 2024. A total of 198 patients were screened and 144 patients were included (73%) as planned by the target sample size. A total of 121 patients (84%) were included at center A, 22 (15%) at center B, and 1 at center C. Seventy-one patients were randomized in the ON group and 71 in the supplemental EN group (1 patient at center A was operated in another hospital after being included and 1 patient was excluded for administrative reason at center C). In total, 24 patients (17%) dropped out for different reasons (18 due to unresectable disease, 3 wanted to stop the study, 1 for failure to put the NJT, 1 received EN preoperatively, and 1 was lost to follow-up), leaving 118 patients for analysis (59 in the enteral group and 59 in the oral group). The study flowchart (Figure 1) summarizes the screening and inclusion process in detail.

The patient characteristics of both groups are summarized in Table 1. Twelve patients (20%) in the ON group and 17 patients (29%) in the supplemental EN group received neoadjuvant chemotherapy.

**Table 1.** Preoperative Characteristics and Intraoperative Details of Included Patients

Characteristic	No. (%)			*P* value
	Oral nutrition group (n = 59)	Enteral nutrition group (n = 59)	Mean difference (95% CI) or RR (95% CI)	
Age, y, median (IQR)	70 (65 to 77)	67 (62 to 75)	−0.8 (−4.3 to 2.8)	.68
Sex				
Women	23 (39)	28 (47)	0.82 (0.5 to 1.2)	.35
Men	36 (61)	31 (53)	1.16 (0.8 to 2.0)	
BMI,^a^ median (IQR)	26 (22.5 to 28.5)	25 (21 to 28)	−0.2 (−1.8 to 1.5)	.86
Neoadjuvant treatment	12 (20)	17 (29)	0.71 (0.4 to 1.3)	.29
Diabetes	15 (25)	11 (19)	1.36 (0.7 to 2.7)	.37
Active smoking	14 (24)	10 (17)	1.40 (0.7 to 2.9)	.36
Jaundice (clinical diagnosis before biliary drainage, if performed)	37 (63)	35 (59)	1.06 (0.8 to 1.5)	.57
Preoperative biliary drainage	35 (59)	34 (58)	1.03 (0.8 to 1.4)	.71
Nutritional risk screening, Kondrup score				
3	27 (46)	26 (44)	NA	.27
4	18 (31)	20 (34)		
5	9 (15)	9 (15)		
6	5 (8)	4 (7)		
ASA score				
I/II	38 (64)	38 (64)	1.0 (1.0 to 1.0)	1.00
III/IV	21 (36)	21 (36)	NA	
Day before surgery prealbumin value, g/L, median (IQR)	0.22 (0.18 to 0.26)	0.22 (0.20 to 0.25)	−0.1 (−0.1 to 0.1)	.16
Highest preoperative CA 19-9 during the month prior the operation, U/mL, median (IQR)	54 (14 to 277)	68 (10 to 494)	−1296 (−4539 to 1946)	.42
Etiologies				
PDAC	27 (46)	28 (47)	NA	.97
Cholangiocarcinomas	12 (21)	11 (19)		
Ampullary carcinomas	11 (19)	8 (14)		
IPMN	3 (5)	3 (5)		
PNET	2 (3)	4 (7)		
Duodenal tumors	2 (3)	2 (3)		
Serous cystadenoma	2 (3)	2 (3)		
Chronic pancreatitis	0	1 (2)		
Pylorus-preserving PD	1 (2)	1 (2)	1.0 (1.0 to 1.0)	1.00
Classic PD	58 (98)	58 (98)	NA	NA
Operative time, min, median (IQR)	370 (322 to 412)	363 (310 to 419)	−2.4 (−38.7 to 34.0)	.90
Intraoperative blood loss, mL, median (IQR)	500 (300 to 800)	500 (300 to 700)	10.6 (−115.3 to 136.5)	.87
Pancreatic texture^b^				
Hard	25 (42)	22 (37)	1.14 (0.8 to 1.8)	.49
Soft	30 (51)	32 (54)		
Size of main pancreatic duct, mm, median (IQR)	4 (3 to 6)	4 (3 to 6)	0.3 (−1.2 to 1.8)	.72
Portal vein resection	2 (3)	3 (5)	0.66 (0.1 to 3.8)	.65

Abbreviations: ASA, American Society of Anesthesiologists; BMI, body mass index; CA, carbohydrate antigen; IPMN, intraductal papillary and mucinous neoplasm; NA, not applicable; PD, pancreatoduodenectomy; PDAC, pancreatic ductal adenocarcinoma; PNET, pancreatic neuroendocrine tumor; RR, risk ratio.

^a^ Calculated as weight in kilograms divided by height in meters squared.

^b^ Data were missing in 4 patients in the oral group and in 5 patients in the enteral group.

The main indications for PD were ductal adenocarcinomas (25 in the ON group and 28 in the EN group), cholangiocarcinomas (10 in the ON group and 11 in the EN group), ampullary carcinomas (11 in the ON group and 8 in the EN group), and intraductal pancreatic mucinous neoplasms (3 in the ON group and 3 in the EN group).

Regarding the main outcome, patients with early supplemental EN had a significantly lower mean (SD) 90-day CCI compared with patients with ON (25.5 [21.1] vs 35.8 [25.2]; mean difference, 10.3; 95% CI, 1.8-18.8; *P* = .02). Morbidity rates at 90 days were 45 of 59 (76%) and 51 of 59 (86%) in the EN and ON groups (RR, 1.13, 95% CI, 0.9-1.9, *P* = .18), respectively. The rate of major complications was 16 of 59 (27%) in the EN group and 26 of 59 in the ON group (44%; RR, 1.62; 95% CI, 1.0-2.7; *P* = .06). No difference in terms of specific complications (DGE, pancreatic fistula, hemorrhage, SSI) was found between the EN and ON groups. Infectious and pulmonary complications were more frequent in the ON group (37% vs 20%; RR, 1.83; 95% CI 1.0-2.6; *P* = .04 and 19% vs 5%, RR, 3.66; 95% CI, 1.1-12.5; *P* = .02). Complications and postoperative outcomes are shown in detail in Table 2.

**Table 2.** Postoperative Results (Primary and Secondary Outcomes) of Included Patients

Outcome	No. (%)			*P* value
	Oral nutrition group (n = 59)	Enteral nutrition group (n = 59)	Mean difference (95% CI) or RR (95% CI)	
Primary outcome				
90-d CCI, mean (SD)	35.8 (25.2)	25.5 (21.1)	10.3 (1.8 to 18.8)	.02
Secondary outcomes				
Overall 90-d morbidity	51 (86)	45 (76)	1.13 (0.9 to 1.9)	.18
Minor complications (1-2)	25 (42)	29 (49)	0.86 (0.6 to 1.3)	.46
Major complications (3-5)	26 (44)	16 (27)	1.63 (1.0 to 2.7)	.06
90-d mortality	1 (2)	1 (2)	1.0 (0.1 to 15.3)	1.00
DGE	24 (41)	29 (4)	0.83 (0.6 to 1.2)	
Grade A	9	13		.36
Grade B	9	7		
Grade C	6	9		
Pancreatic fistula	22 (37)	16 (27)	1.38 (0.8 to 2.3)	
Grade A	13	12		.24
Grade B	7	3		
Grade C	2	1		
Hemorrhage	7 (12)	7 (12)	1.0 (0.4 to 2.7)	
Grade A	2	2		1.00
Grade B	2	2		
Grade C	3	3		
SSI	20 (34)	14 (24)	1.43 (0.8 to 2.6)	
Superficial	2	2		.22
Deep	1	1		
Organ space	17	11		
Infectious complications^a^	22 (37)	12 (20)	1.83 (1.0 to 2.6)	.04
Pulmonary complications	11 (19)	3 (5)	3.66 (1.1 to 12.5)	.02
Length of stay, d, median (IQR)^b^	17 (14 to 28)	19 (13 to 27)	−1.9 (−8.5 to 4.7)	.56
90-d readmission	13 (22)	16 (27)	0.81 (0.4 to 1.5)	.52

Abbreviations: CCI, Comprehensive Complication Index; DGE, delayed gastric emptying; SSI, surgical site infection; RR, risk ratio.

^a^ Excluding SSIs.

^b^ Length-of-stay data were missing for 12 patients (5 in the oral group and 7 in the enteral group).

Thirty patients (51%) in the EN group required PN during the postoperative period and 32 patients (54%) in the ON group (*P* = .71). Time to reach 50% of total caloric requirements with ON was similar in both groups (ON group: median [IQR] 5 [3-13] days vs EN group: 6 [4-12] days; *P* = .34).

**Figure 2.**
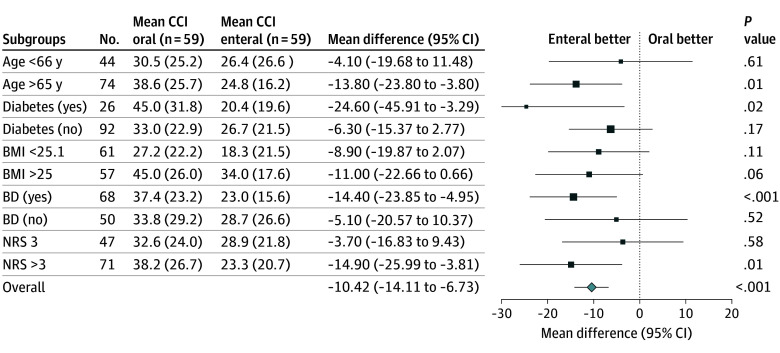
Subgroup Analyses of the Primary Outcome (Comprehensive Complication Index [CCI]) BD indicates biliary drainage; BMI, body mass index (calculated as weight in kilograms divided by height in meters squared); NRS, nutritional risk screening.

Patients older than 65 years old (mean difference, −13.8; 95% CI, −23.8 to −3.8; *P* = .01), with diabetes (mean difference, −24.6; 95% CI, −45.9 to −3.3; *P* = .02), who had a preoperative biliary drainage (mean difference, −14.4; 95% CI, −23.9 to −5.0; *P* < .01), and with an NRS score more than 3 (mean difference, −14.9; 95% CI −26.0 to −3.8; *P* = .01) had significantly higher mean CCI. If the Bonferroni adjusted *P* value is used (1%), diabetes is no longer significant. Details of the subgroup analysis can be found in Figure 2.

Three patients allocated to the EN group did not receive EN during hospitalization due to mechanical obstructive problems with the NJT (n = 2) and failure of NJT insertion (n = 1). For the per-protocol analysis, these 3 patients were taken into account in the ON group, leaving 56 patients in the EN group and 62 patients in the ON group. Mean CCI in the per protocol cohort was significantly higher in the ON group (mean difference, 11.4; 95% CI, 2.9-19.8; *P* = .009). Details can be found in the eResults in Supplement 2.

If perioperative deaths (n = 2, 1 in each group) are excluded from the analysis of the primary end point, mean CCI remains lower in the enteral group (mean difference, 10.5; 95% CI, 2.9-18.8; *P* = .08) (eResults in Supplement 2).

In the EN group (n = 59), 14 patients (24%) involuntarily removed their NJT during the postoperative period (accidentally or during vomiting) and required NJT replacement. The median (IQR) duration of EN was 10 (7-16) days. Ten patients mentioned slight to moderate discomfort (>2 on a 0-10 scale) during EN on postopertive day 3. Median (IQR) flow rate of enteral nutrition on postopreative day 3 was 30 (21-42) ml per hour. Ten patients were discharged with EN that was continued at home.

## Discussion

The present RCT compared early supplemental EN and ON with ON alone after PD in patients at increased nutritional risk. Patients receiving early supplemental EN postoperatively had lower CCI than patients receiving only ON, indicating that patients with EN presented a decreased burden of complications.

A recent propensity–score matched study on 428 patients comparing EN with ON after PD found lower rates of grade B and C DGE in patients with ON, while the overall morbidity was not impacted.^23^ At first glance, the findings are in contrast to the present trial showing benefits of early EN. It must be noted that the study by Jing et al^23^ included patients irrespective of their nutritional status, while in the NUTRIWHI trial^14^ only patients with an NRS score of 3 or more were included. Moreover, decompressive NGT were routinely used in their study during the first day postoperative, while NGT were removed at the end of surgery in the present study. Lastly, propensity–score matching studies are associated with intrinsic limitations that preclude full comparison with RCTs.

A French multicentric RCT including 9 centers compared EN with PN after PD.^9^ The authors found that patients with PN had a lower rate of postoperative complications (64.4% vs 77.5%; *P* = .04). Moreover, EN was associated with more frequent and more severe pancreatic fistulas. A meta-analysis of 5 RCTs found no difference in terms of complications between EN and PN following PD.^24^ Only the LOS was shorter in patients with EN (weighted mean difference, −1.63 days; 95% CI, −2.80 to −0.46; *P* = .006). Similarly, a meta-analysis published by Cai et al^25^ in 2020 included 9 studies comparing EN with PN and found a shorter LOS after EN but similar complication rates. A meta-analysis by Tanaka et al^26^ showed that percutaneous EN had a lower rate of infectious complications, as well as shorter LOS compared with PN after PD (3 RCTs), while no difference was found comparing EN via NJT with PN (2 RCTs). In the present study, the aim was to compare ON with early EN in addition to ON. As several guidelines recommend ON postoperatively after PD,^7,8^ the purpose of this trial was to assess whether adding EN to ON in malnourished patients lead to less postoperative complications. To avoid bias with the use of PN, a standardized definition of when to start PN was applied similarly to both groups (EN and ON).

EN was well tolerated by most patients (49 of 59). The fact that the NJT was placed during surgery was an advantage. One drawback of the NJT is that it can be withdrawn unintentionally rather easily. This was one unexpected finding of the present results (14 unintentional tube removals in the EN group needing tube replacement).

The lower rates of infectious and pulmonary complications in the enteral group contributed to the decreased total burden of complications (CCI) in the enteral group. EN might potentially have improved the body capacity to respond to an infectious trigger and avoided development of a clinically relevant infection. Another potential explanation of why EN was better than ON in our study might be the fact that patients with DGE (45% in the entire cohort), needing a decompression NGT, benefited from intestinal stimulation with the EN rather than just having PN. Moreover, as patients with EN reached the required caloric levels faster than patients with ON only, an improved caloric intake could have had a positive impact on postoperative results. It can also be mentioned that there were slightly more smokers, patients with diabetes, and patients with jaundice included in the ON group, even though not significant.

Several questions remain unanswered regarding perioperative nutrition in patients undergoing PD. Are oral nutritional supplements necessary in patients without malnutrition? Additionally, what threshold and which score should be used to identify patients at increased nutritional risk and who should benefit from preoperative enteral or parenteral nutritional support? Regarding postoperative nutrition, if one considers EN, the best way of delivering it (NJT vs surgical or percutaneous tube) is still unknown. This study paves the way for further research in this field and gives perspectives for future trials on perioperative nutrition in patients undergoing PD. In the context of ERAS and early resumption of ON postoperatively, the findings of this study could bring more arguments to recommend early supplemental EN for patients nutritionally at risk. Subgroup analyses of the primary outcome showed that in subpopulations of patients older than 65 years, patients with diabetes, patients with preoperative biliary drainage, and patients with an NRS score higher than 3, the CCI was lower in the EN group. These subgroup analyses generate hypotheses that EN might be more beneficial in these aforementioned patient cohorts. Yet, these results should be validated by future prospective studies as the sample size of the present study was not calculated to have enough power for subgroup analyses and these analyses have a risk of multiple testing bias.

### Strengths and Limitations

The strengths of this study are its randomized design and the standardized protocol for ON, EN, and PN. A 30% decrease of the mean CCI as found in this study has clinical relevance as it positively impacts the outcomes of several patients.

Some limitations of this study need to be mentioned. The dropout rate (17%) might be a risk for selection bias. To assess this potential risk, characteristics of excluded and included patients were compared and analyses adjusting for covariates were performed. Even though all included centers used an ERAS pathway for perioperative care and the perioperative nutrition program was standardized, some heterogeneity might persist. The sample size was relatively small but was based on the power calculation to reach a potential 30% CCI difference. This targeted sample size was respected. The patient recruitment showed a large variability as one center included 80% of all patients, which played a dominant role in the results. Even though center-adjusted analyses were performed (eFigure 1 in Supplement 2), it should be kept in mind that these analyses might not be well powered as number of centers and minimal number per center were not included in the sample size calculation. Blinding was not possible for patients and surgeons, which can be a cause of bias. The heterogeneity of the indications (histologies) and the high rate of PN might limit the generalizability of the findings. As only patients who are the most at risk nutritionally were included, the conclusion of this study should not be applied to well-nourished young patients. However, as patients undergoing PD already have 2 points for the severity of the disease (major abdominal operation), most patients will have an NRS score of 3 or more, meaning that the present results could be broadly applied in a population of patients needing a PD. The present study design can be debatable because EN was not allowed in the ON group. The principle to prioritize EN over PN in this specific setting would have been particularly relevant and valuable for patients with low appetite but no DGE. Notwithstanding, the need for artificial nutrition after PD mostly occurs in patients with DGE. In these patients, the use of EN would have required an endoscopy under sedation/anesthesia to safely place the NJT. This would have been associated with a risk of aspiration pneumonia or injury to the recent gastrojejunal anastomosis (mechanical injuries or due to the insufflation). Lastly, using EN instead of PN for patients in the ON group would have been an interesting option that was not considered herein, in particular for patients without DGE.

## Conclusions

In conclusion, in patients with an NRS score of 3 or more undergoing PD, this RCT found that early supplemental EN in addition to ON was well tolerated and decreased the burden of postoperative complications compared with ON alone.

## Notes

Supplement 1. Study protocol

Supplement 2. eMethods Enteral and oral nutrition protocols Perioperative management Study protocol and role of the funding sources  Definitions Statistical analyses eTable 1. Comparisons between excluded and included patients eResults. Sensitivity analyses eFigure 1. Subgroup analyses and Kaplan-Meier graph eTable 2. Complementary analyses on main outcome eReferences

Supplement 3. Data sharing statement
