# Supplementary material for: Early Enteral vs Oral Postoperative Nutrition After Pancreatoduodenectomy: The NUTRIWHI Randomized Clinical Trial
Source: JAMA Surg. 2026 Apr 22;161(6):575–82. doi: 10.1001/jamasurg.2026.1048 (PMC13103875; doi:10.1001/jamasurg.2026.1048)
Supplement: Supplement 1. — Study protocol [file jamasurg-e261048-s001.pdf]

Service de chirurgie viscérale
Prof. N. Demartines, Chef de Service
Rue du Bugnon 46 / BH15 nord
CH-1011 Lausanne

Early enteral *vs*. oral nutrition after Whipple procedure: a multicentric randomized controlled trial

NUTRIWHI Trial Clinical Study Protocol

Study Type:	Other Clinical Trial according to ClinO, Chapter 4
Risk Categorisation:	Risk category A according to ClinO, Art. 61
Study Registration:	Intended registries: ClinicalTrials.gov, SNCTP (Swiss National Clinical Trial Portal)
Sponsor:	Centre hospitalier universitaire vaudois, CHUV (bpr@chuv.ch)
Sponsor representative:	Prof. Markus Schäfer<br>Department of Visceral Surgery<br>Centre hospitalier universitaire vaudois, CHUV, Lausanne
Principal Investigator:	Dr Gaëtan-Romain Joliat<br>Department of Visceral Surgery<br>Centre hospitalier universitaire vaudois, CHUV, Lausanne
Investigated Intervention:	Enteral nutrition compared to oral nutrition after Whipple procedure
Protocol ID	2021-00724
Version and Date:	Version 5.0 (dated 20/10/2023)

CONFIDENTIALITY STATEMENT
The information contained in this document is confidential and the property of the sponsor. The information may not - in full or in part - be transmitted, reproduced, published, or disclosed to others than the applicable Competent Ethics Committee(s) and Regulatory Authority(ies) without prior written authorisation from the sponsor except to the extent necessary to obtain informed consent from those who will participate in the study.

Principal Investigator

Name: Dr Gaëtan-Romain Joliat

Sponsor representative

Name: Prof. Markus Schäfer

Local Principal Investigators at study sites:

Site:

Hôpital Cochin – Port Royal, AP-HP
27, rue du Faubourg Saint-Jacques
75014, Paris, France

Principal Investigator: Dr Ugo Marchese

Site:

Regional Hospital of Lugano
Via Tesserete 46
6900 Lugano, Ticino, Switzerland

Principal Investigator: Dr Alessandra Cristaudi

Table of Contents

GLOSSARY OF ABBREVIATIONS 5
1 STUDY SYNOPSIS 6
2 BACKGROUND AND RATIONALE 10
3 STUDY OBJECTIVES AND DESIGN 11
3.1 Hypothesis and primary objective 11
3.2 Primary and secondary endpoints 12
3.3 Study design 13
3.4 Study intervention 13
4 STUDY POPULATION AND STUDY PROCEDURES 14
4.1 Inclusion and exclusion criteria, justification of study population 14
4.2 Recruitment, screening and informed consent procedure 15
4.3 Study procedures 15
4.4 Withdrawal and discontinuation 17
5 STATISTICS AND METHODOLOGY 17
5.1. Statistical analysis plan and sample size calculation 17
5.2. Handling of missing data and drop-outs 19
6 REGULATORY ASPECTS AND SAFETY 19
6.1 Local regulations / Declaration of Helsinki 19
6.2 Serious Adverse Events and notification of safety and protective measures 19
6.3 (Periodic) safety reporting 21
6.4 Radiation 21
6.5 Amendments 21
6.6 (Premature) termination of study 21
6.7 Insurance 22
7 FURTHER ASPECTS 22
7.1 Overall ethical considerations 22
7.2 Risk-benefit assessment 22
8 QUALITY CONTROL AND DATA PROTECTION 23
8.1 Quality measures 23
8.2 Data recording and source data 23
8.3 Confidentiality and coding 23
8.4 Retention and destruction of study data and biological material 24
9 MONITORING AND REGISTRATION 24
10 FUNDING / PUBLICATION / DECLARATION OF INTEREST 24
11 REFERENCES 25
Appendix 1: Schedule of assessments 29

GLOSSARY OF ABBREVIATIONS

AE	*Adverse Event*
ASR/DSUR	*Annual Safety Repot / Development Safety Report*
BASEC	*Business Administration System for Ethical Committees*
BIA	*Bioelectrical Impedance Analysis*
BMI	*Body-Mass Index*
CCI	*Comprehensive Complication Index*
CHUV	*Centre hospitalier universitaire vaudois*
ClinO	*Ordinance on Clinical Trials in Human Research (in German: KlinV, in French: OClin, in Italian: OSRUm)*
CRC	*Clinical Research Center*
CRF	*Case Report Form*
CRP	*C-Reactive Protein*
CTCAE	*Common Terminology Criteria for Adverse Events*
DGE	*Delayed Gastric Emptying*
eCRF	*electronic Case Report Form*
EEN	*Early Enteral Nutrition*
EORTC	*European Organization for Research and Treatment of Cancer*
ERAS	*Enhanced Recovery After Surgery*
ESPEN	*European Society for Parenteral and Enteral Nutrition*
FADP	*Federal Act on Data Protection (in German: DSG, in French: LPD, in Italian: LPD)*
FOPH	*Federal Office of Public Health*
GCP	*Good Clinical Practice*
HRA	*Human Research Act (in German: HFG, in French: LRH, in Italian: LRUm)*
ICH	*International Conference on Harmonisation*
NRS	*Nutritional Risk Screening*
PAC	*Pancreatic Cancer*
PD	*Pancreatoduodenectomy*
POD	*Postoperative Day*
POPF	*Postoperative Pancreatic Fistula*
PPH	*Postpancreatectomy Hemorrhage*
QoL	*Quality of Life*
RCT	*Randomized Controlled Trial*
REDCap	*Research Electronic Data Capture*
SAE	*Serious Adverse Event*
SSI	*Surgical Site Infection*
TPN	*Total Parenteral Nutrition*

1 STUDY SYNOPSIS

**Principal Investigator**	Dr Gaëtan-Romain Joliat, Visceral Surgery, Centre hospitalier universitaire vaudois, CHUV
**Sponsor and sponsor representative**	Centre hospitalier universitaire vaudois, CHUV<br>Prof. Markus Schäfer, Visceral Surgery, Centre hospitalier universitaire vaudois, CHUV
**Study Title**	Early enteral *vs.* oral nutrition after Whipple procedure: a multicentric randomized controlled trial
**Short Title / Study ID**	NUTRIWHI Trial / 2021-00724
**Protocol Version and Date**	Version 5 (dated 20/10/2023)
**Study Registration**	ClinicalTrials.gov and Swiss National Clinical Trials Portal (SNCTP)
**Study Category and Rationale**	Other Clinical Trial (ClinO, Chapter 4)<br>Category A (Art. 61): The health-related intervention (enteral nutrition) of this clinical trial entails only minimal risks and constraints for the patients.
**Background and Rationale**	Pancreatic cancer (PAC) is one of the deadliest cancers in humans (more than 47'000 estimated deaths in 2020 in the United States) and the most frequent indication for pancreatoduodenectomy (PD)[1]. It is predicted to become the second most common cause of cancer deaths in the United States by 2030[2]. The current observed 3-year overall survival after diagnosis is 6%[3]. Even if operated, pancreas cancer has a bad long-term prognosis (5-year overall survival 20-30%)[4]. The majority of PAC types are adenocarcinomas and approximately 60-70% of them are localized in the head of the pancreas[4]. To date, surgery remains the only potentially curative strategy. However, PD remains a difficult surgical procedure embedded with high morbidity (40-60%) even though progresses have been made in surgical techniques and anesthesia[5,6].<br>Patients suffering from PAC as well as patients with chronic pancreatitis or requiring pancreas surgery often are in a compromised nutritional status[7]. Nutritional support should therefore be started early during the postoperative course to prevent further malnutrition, as it is an important risk factor to develop complications[8–10]. Recently, several studies have shown that early enteral nutrition (EEN) could shorten length of stay, reduce postoperative infections and mortality, and decrease costs when compared with total parenteral nutrition (TPN) in gastrointestinal cancer surgery[11–14]. After PD, EEN has been shown to reduce early and late complications, infections, and readmission rates[15]. It is nevertheless currently not clear if EEN improves the short-term outcomes after PD compared to oral nutrition.
**Risk / Benefit Assessment**	There are several adverse effects associated with nasojejunal tube and enteral nutrition: poor tolerance, nausea, vomiting, diarrhea, tube obstruction, or bronchial inhalation. Nursing teams are nevertheless trained to use this equipment and will perform the same care as usual: position verification, tube flushing, nasal fixation, and nasal eschar surveillance. With these measures, the risk of adverse events associated with the study is estimated to be low.<br>From the investigators' perspective, the EEN intervention will be a benefit for patients included in the study group. Nevertheless, the control group cannot be considered unfavored as currently no intervention of this type exists in visceral surgery and oral nutrition is presently given after PD.
**Objectives**	1. Impact of EEN on postoperative morbidity after PD, according to the Comprehensive

	Complication Index (CCI)[16].<br>2. Impact of EEN on major complications, according to Clavien classification[17], specific postoperative complications, length of stay, readmission rates, quality of life, metabolic stress and nutritional response after PD.
**Endpoints**	1. Primary outcome: postoperative morbidity, as evaluated according to the CCI within 90 postoperative days.<br>2. Secondary endpoints:<br>• Major postoperative complications, as evaluated according to the Clavien classification within 90 postoperative days (Clavien grade >II).<br>• Specific complications after PD will be recorded:<br>o Surgical site infections (SSI)[18]<br>o Postoperative pancreatic fistula (POPF)[19]<br>o Delayed gastric emptying (DGE)[20]<br>o Postpancreatectomy hemorrhage (PPH)[21]<br>o Biliary fistula<br>o Gastrojejunal anastomosis fistula<br>o Pancreatitis<br>• Length of stay will be quantified from operative day until discharge.<br>• Readmissions will be assessed from day 1 after discharge until postoperative day 90.<br>• Patients quality of life (QoL) will be assessed by the EORTC (European Organisation for Research and Treatment of Cancer) QLQ-C30 questionnaire[22].<br>• Metabolic response to EEN will be assessed with biological measurements preoperatively and twice weekly during hospital stay (currently already measured according to our PD care map):<br>o C-Reactive Protein (CRP) and procalcitonin<br>o Simple blood count, coagulation tests<br>o Electrolytes: sodium, potassium, calcium, magnesium, phosphate<br>o Creatinine, urea, blood glucose, liver and pancreatic function tests, prealbumin, albumin, triglycerides<br>• Assessment of malabsorption due to PD with biological measurements preoperatively and between the 4th and 6th postoperative week:<br>o Folate, iron, ferritin, transferrin saturation<br>o Vitamin D, parathormone, vitamin E<br>o Glycated hemoglobin (HbA1c)<br>• Measure of lean body mass using bioelectrical impedance analysis and of muscle strength based on the handgrip preoperatively, on discharge day, and on the first follow-up visit.<br>• Measure of resting energy expenditure using indirect calorimetry on postoperative day 5.
**Study Design**	Multicentric, open-label, two-arm, randomized controlled trial (balanced groups 1:1)

**Statistical Considerations**	We will use a Student's *t*-test to evaluate if the primary outcome (CCI) can significantly be reduced by EEN compared to the control group. For the analysis of all secondary endpoints, we will also use *t*-tests or chi-squared tests depending on the variable types.<br>The analysis will be based on the "intention to treat" model. An explanation on sample size is given hereunder (see "Number of Participants with Rationale").
**Inclusion / Exclusion Criteria**	Inclusion criteria<br>o Patient scheduled for elective open PD.<br>o Patient ≥18 years old.<br>o Patient at nutritional risk, i.e., Nutritional Risk Screening (NRS) ≥3[23].<br>o Signed informed consent.<br>Exclusion criteria<br>o Patient not able to give consent (e.g., vulnerable patient).<br>o Enteral feeding already initiated preoperatively.<br>o Language barrier.<br>o Inability for the participant to follow the procedures of the study: language problems, psychological disorders (eating and bipolar disorders) or dementia.
**Number of Participants with Rationale**	According to a recent randomized trial including a series of PD and assessing a realimentation process (enteral *vs*. parenteral), the mean CCI was impacted by about 30% (32.8 vs. 24.2). Another study reported a mean CCI of 38 after PD[24]. We hypothesized that ENN would reduce a mean CCI of 35 (± 20) by 30%.<br>Assuming a CCI reduction of 10.5 points (30% of 35), a standard deviation of ± 20, a 80% power, an alpha of 5% and a dropout rate of 20%, the total required number of patients is 144 (72 study patients = EEN group and 72 control patients = oral nutrition group).
**Study Intervention**	In our current practice, no feeding or suction gastric tube is left in place after PD. Patients will receive standardized perioperative care according to the Enhanced Recovery After Surgery protocol[25]. A standard nutrition protocol for the EEN intervention group has been established in accordance with the nutritionists:<br>The night after the operation, a low flow enteral nutrition will be initiated (21 ml/h, 250 ml/12 h), and based on an Isosource® Energy Fibre solution (hypercaloric).<br>If the tolerance is subjectively good, with a visual analogue scale ≤4/10, the flow will be increased on postoperative day (POD) 1, at the flow of 42 ml/h (500 ml/12 h).<br>On POD 2: increased flow to 62.5 ml/h (750 ml/12 h).<br>On POD 3: increased flow to 83.5 ml/h (1000 ml/12 h).<br>If the tolerance is not satisfactory (>4/10), the current flow will be decreased, maintained 24 hours more or put on hold for a couple of hours according to the symptoms of the patient.<br>The nutrition will be infused over 12 hours with a pump and controlled flow rate. Enteral nutrition will be continued until oral food intake reaches 50% of nutritional requirements defined as 30 kcal/kg/day for patients with BMI <30 kg/m^2 or 25 kcal/kg/day for patients with BMI ≥30 kg/m^2 (oral intake will be assessed by the dietician).

**Control Intervention**	The control group will receive oral nutrition after the operation as it is presently done in our division based on our clinical care maps.
**Study procedure**	At the end of surgery, a polyurethane single or double-lumen feeding nasojejunal tube (Freka®) 8 French will be placed under direct visual control, 30 cm distally to gastrojejunostomy, in patients randomized to EEN. The tube will be attached to the nose wing with a tape according to current institutional practice.
**Study Duration and Schedule**	Three years<br>Planned date of first participant-in: October 2021<br>Planned date of last-participant-out: October 2024
**Main Investigators**	Dr Gaëtan-Romain Joliat, Visceral Surgery, Centre hospitalier universitaire vaudois, CHUV<br>Dr David Martin, Visceral Surgery, Centre hospitalier universitaire vaudois, CHUV<br>Dr Ismail Labgaa, Visceral Surgery, Centre hospitalier universitaire vaudois, CHUV<br>Prof. Nicolas Demartines, Visceral Surgery, Centre hospitalier universitaire vaudois, CHUV<br>Prof. Markus Schäfer, Visceral Surgery, Centre hospitalier universitaire vaudois, CHUV
**Study Centers**	Centre hospitalier universitaire vaudois, CHUV<br>Rue du Bugnon 21<br>1011 Lausanne, Vaud, Switzerland<br><br>Hôpital Cochin – Port Royal, AP-HP<br>27, rue du Faubourg Saint-Jacques<br>75014 Paris, France<br><br>Regional Hospital of Lugano<br>Via Tesserete 46<br>6900 Lugano, Ticino, Switzerland
**Data privacy**	The investigators affirm and uphold the principle of the participant's right to privacy and that they will comply with applicable privacy laws. Especially, anonymity of the participants will be guaranteed when presenting the data at scientific meetings or publishing them in scientific journals.<br><br>Individual subject medical information obtained as a result of this study is considered confidential and disclosure to third parties is prohibited. Subject confidentiality will be further ensured by utilizing subject identification code numbers to correspond to treatment data in the computer files.
**Ethical consideration**	The goal of this study is to define the best route for nutrition after PD. The method of randomization will permit to have a high level of evidence.<br>There are several adverse effects associated with nasojejunal tube and enteral nutrition as mentioned above in the risk-benefit section. Nurses and doctors of our division are nevertheless used to this equipment, frequently used in visceral surgery. The risk of adverse events associated with the study is therefore judged as low.<br>From the investigators' perspective, the EEN intervention is hypothesized to benefit patients. Nevertheless, the control group cannot be considered as unfavored as the current nutritional management consists of oral nutrition.<br>No vulnerable populations will be included.

**GCP Statement**	This study will be conducted in compliance with the protocol, the current version of the Declaration of Helsinki, the ICH-GCP, the HRA as well as other locally relevant legal and regulatory requirements.

2 BACKGROUND AND RATIONALE

Pancreatic cancer (PAC) is one of the deadliest cancers in humans (more than 47'000 estimated deaths in 2020 in the United States)[1]. It is predicted to become the second most common cause of cancer deaths in the United States by 2030[2]. The mean costs in 2015 were estimated to be $79'800 per patient with PAC and $164'100 for each resection[26]. The observed overall 3-year survival after diagnosis is 6%[3]. The majority of PAC are adenocarcinomas and approximately 60-70% of them are localized in the head of the pancreas[4]. To date, surgery remains the only potentially curative strategy. However, resection of the pancreatic head remains a difficult surgical procedure embedded with high morbidity (40-60%)[5,6]. Recently, the concept of Enhanced Recovery After Surgery (ERAS) has contributed to reduce overall morbidity, length of hospital stay and costs by implementing multimodal measures influencing the pre-, intra- and postoperative periods[27–31].

Patients suffering from pancreatic tumors as well as patients with chronic pancreatitis often present with cachexia or at least with a certain level of malnutrition[7]. This situation is difficult to correct preoperatively due to the pressure to operate these patients rapidly. Nutritional therapy should therefore be started early during the postoperative course to prevent further malnutrition, as the latter is an important risk factor to develop complications[8–10]. This is even more relevant for patients with poor preoperative nutritional status. In addition, surgery disrupts the digestive tract, leading to postoperative indigestion and malabsorption[32]. Postoperative nutritional supports, including early enteral nutrition (EEN) and total parenteral nutrition (TPN), have been shown to be effective in improving clinical outcomes after major abdominal surgery[8].

Malnutrition is still poorly defined. Many definitions have been proposed based on criteria that vary between medical history, biometric and biological data. Currently, the European Society for Parenteral and Enteral Nutrition (ESPEN) recommends the Nutritional Risk Score (NRS-2002) as a screening tool, even if it has not been prospectively validated[23,33]. Several studies have proven its reliability to identify patients at nutritional risk who will benefit from perioperative nutritional support[9,34]. Patients with NRS ≥3 are considered to be exposed to higher incidence and severity of postoperative complications.

Recent randomized clinical trials and meta-analyses have shown that EEN could shorten length of stay, reduce postoperative infections and mortality and improve cost-effectiveness when compared to TPN in gastrointestinal cancer surgery[11–14]. Specifically after

pancreatoduodenectomy (PD), EEN reduces early and late complications, infections, and readmission rates[15]. Another retrospective study showed no differences with respect to time to resumption of normal oral intake, morbidity and mortality when comparing EEN via nasojejunal tube or jejunostomy tube and total parenteral nutrition[35]. However, a recent multicentric randomized controlled trial compared nasojejunal EEN to TPN after PD. This study showed that EEN was associated with an increase of overall postoperative complications[24]. One major drawback of this study is that it did not compare EEN to the recognized standard which is oral feeding and not TPN[25]. Another systematic review compared the outcomes of 5 feeding routes after PD (oral diet, enteral nutrition via either a nasojejunal, gastrojejunostomy tube or jejunostomy tube, and TPN) and reported no evidence to support routine enteral or parenteral feeding after PD[36].

The study of EEN and its impact in terms of morbidity require the use of a validated tool. Most studies fail to provide information about the severity of complications and inform only on the most severe event, ignoring events of lesser severity[17]. The Comprehensive Complication Index (CCI) was created to summarize all postoperative complications and is more sensitive than existing morbidity endpoints[16].

The primary objective of the study is to assess the impact of EEN on postoperative morbidity after PD, according to the CCI. Secondary objectives are to assess the impact of EEN on major postoperative complications, according to Clavien classification, specific complications, length of stay, readmission rates, quality of life (QoL), metabolic stress and nutritional response after PD.

The risk category for this study is Category A according to ClinO, Art. 61. The health-related intervention submitted to the clinical trial involves only minimal risks and burdens. The risks are linked to the nasojejunal tube (poor tolerance, nausea, …). The investigators evaluate these risks as minimal. Moreover, the nursing staff is used to this type of nasojejunal tube as it is often routinely used in visceral surgery and in our division.

3 STUDY OBJECTIVES AND DESIGN

3.1 Hypothesis and primary/secondary objectives

The hypothesis is that EEN after PD might decrease the postoperative complications compared to oral nutrition as patients undergoing PD often are malnourished. The primary objective is to assess the impact of EEN on postoperative morbidity, according to the CCI, after PD in patients at nutritional risk with a NRS ≥3.

The secondary objective is to evaluate the impact of EEN on major postoperative complication, according to Clavien classification, specific complications, length of stay, readmission rates, QoL, metabolic stress and nutritional response after PD in patients at nutritional risk with a NRS ≥3.

3.2 Primary and secondary endpoints

The primary endpoint measuring postoperative morbidity will be assessed using the CCI within 90 postoperative days.

Secondary endpoints are the following:

- Most severe postoperative complications will be measured using the Clavien classification within 90 postoperative days.
- Specific complications of PD will be recorded:
  - SSI, further divided into 'superficial', 'deep' and 'organ-space' according to the specific anatomic involvement and the Centers for Disease Prevention definition[18].
  - POPF are classified into three grades, A, B and C, according to the consensus of the International Study Group for Pancreatic Surgery (ISGPS)[19].
  - DGE, which is classified into three grades, A, B and C, according to the consensus of the ISGPS[20].
  - PPH, which is also classified into three grades, A, B and C, according to the consensus of the ISGPS[21].
  - Biliary fistula (no standard definition)
  - Gastrojejunal anastomosis fistula (no standard definition)
  - Pancreatitis (no standard definition)
- Length of stay will be measured from operative day until discharge.
- Readmissions will be counted until postoperative day 90.
- Patients' QoL will be assessed by the EORTC (European Organisation for Research and Treatment of Cancer) QLQ-C30 questionnaire[37]. This questionnaire will be filled 4 times: at preoperative consultation or admission, at patient's discharge, between the 4th and 6th postoperative week and on POD 90 (via phone call).
- The subjective tolerance of EEN will be assessed daily during the first 7 postoperative days, using a visual analogue scale (0-10). Objective tolerance will be assessed by the amount of EEN as a percentage (tolerated/total amount of EEN required).
- Time required (in days) to reach respectively 50% and 100% of the daily caloric targets required (30 kcal/kg/day if BMI <30 kg/m^2 and 25 kcal/kg/day if BMI ≥30 kg/m^2, protein target: 1.5 g/kg/day).
- Metabolic response to EEN will be assessed with biological measurements preoperatively and twice weekly (currently already measured, according to our PD care map):
  - C-Reactive Protein (CRP) and procalcitonin
  - Simple blood count, coagulation
  - Electrolytes: sodium, potassium, calcium, magnesium, phosphate
  - Creatinine, urea, blood glucose, liver and pancreatic function tests, prealbumin,

albumin, triglycerides

- Various malabsorption due to PD surgery: measurements will be made twice, once before surgery and once between the 4th and 6th postoperative week during the follow-up visit. As PD might induce duodenal and pancreatic insufficiencies postoperatively due to the resection of the duodenum and the pancreatic head, it is presently unknown if EEN might have an effect on these insufficiencies by improving the overall nutritional state and the mucosal trophicity of the small bowels.
  - Duodenal insufficiency: folate, magnesium, calcium, iron, ferritin, transferrin saturation
  - Exocrine pancreatic insufficiency: malabsorption of fat soluble vitamins: vitamin D (with calcium/phosphate balance and parathormone) and vitamin E
  - Endocrine pancreatic insufficiency: due to risk of developing a secondary diabetes, HbA1c (glycated hemoglobin) will be measured.
- Body measure using bioelectrical impedance analysis (BIA) and muscle strength using handgrip will be measured preoperatively, on discharge day, and on the first follow-up visit.
- Resting energy expenditure will be measured on POD 5 using indirect calorimetry.

Each of these endpoints will be measured in the study (EEN) and control (oral nutrition) groups.

3.3 Study design

This study is an open-label, multicentric, international two-arm, randomized controlled trial.

The downside of the selected design (RCT) will be the study duration (i.e., three years). The randomization will permit to minimize biases by having two comparable groups.

3.4. Study intervention

After PD, patients will be randomized to receive either EEN or oral nutrition. Patients included in the EEN arm will receive, in addition to oral nutrition based on the current care maps, enteral nutrition according to the following scheme that was established in accordance with nutritionists:

- Six hours after the operation, a low flow enteral feeding will be initiated (21 ml/h, 250 ml/12h), and based on a Isosource® Energy Fibre solution (hypercaloric, 400 kcal).
- If the tolerance is subjectively good, with a visual analogue scale ≤4/10, the flow will be increased on first postoperative day (POD), at the flow of 42 ml/h (500 ml/12h, 800 kcal).
- On POD 2: increased flow to 62.5 ml/h (750 ml/12h, 1200 kcal)
- On POD 3: increased flow to 83.5 ml/h (1000 ml/12h, 1600 kcal)

If the tolerance is not satisfactory (>4/10), the current flow will be maintained 24 hours more, decreased or put on hold for a couple of hours (depending on the symptoms of the patient), and increased the next day if tolerated until the maximum of 1000 ml/12h.

The diet will be infused over 12 hours with a pump and controlled flow rate. EEN will be continued

until oral food intake will have reached more than 50% of nutritional requirements. Daily nutritional requirements will be defined as 30 kcal/kg if BMI <30 kg/m^2 and 25 kcal/kg if BMI ≥30 kg/m^2 (protein target: 1.5 g/kg/day). The oral intake will be assessed by the dietitian.

If a patient in the EEN group loses or displaces its nasojejunal tube (vomiting, accidental removal), a new probe will be replaced under endoscopic control by the gastroenterologist. If that happens a second time, another attempt to put the nasojejunal tube will not be made (the patient will remain in the study). In the same previous scenario (nasojejunal tube expulsion), and if the patient suffers from DGE, a nasogastric tube will be installed at the same time. If the patient suffers from DGE and the nasojejunal tube is in place, a nasogastric suction tube will be installed in addition, and enteral feeding will be continued. Parenteral nutrition will be used to complete the missing caloric needs. In the control group (without nasojejunal tube), in case of DGE, a nasogastric suction tube will be installed (the patient will remain in the study).

The use of parenteral feeding will be standardized similarly in both groups. A parenteral nutrition will be initiated if the caloric intake is <50% of caloric requirements for 24 hours and from POD 3. Parenteral nutrition will be continued until the total caloric intake without the parenteral nutrition reach >50% of daily caloric needs and until no more nasogastric tube will be in place.

Note that according to the current standard care, endoscopies are also performed in case of repeated vomiting, DGE, loss of feeding tube or to put a triluminal probe. No additional endoscopies will be performed as study procedures.

4 STUDY POPULATION AND STUDY PROCEDURES

4.1 Inclusion and exclusion criteria, justification of study population

Participants fulfilling all of the following inclusion criteria are eligible for the study:

- Patient scheduled for elective open PD.
- Patient ≥18 years old.
- Patient at nutritional risk, i.e., with NRS ≥3.
- Signed informed consent

The presence of any one of the following exclusion criteria will lead to exclusion of the participant:

- Patient not able to give informed consent as documented by signature of consent form (e.g., vulnerable patients).
- Enteral feeding already initiated preoperatively.
- Language barrier.
- Inability to follow the procedures of the study, e.g., due to language problems, psychological disorders (i.e., eating disorders and bipolar disorders), or dementia.

The total number of included patients will be 144 (72 in each group, see section 5.0 for further details). The choice of the patient population is justified by the fact the patients undergoing PD often are malnourished (cachexia due to cancer or chronic pancreatitis) and are at nutritional risk postoperatively due to the important stress response induced by this major abdominal surgery. As malnutrition is a risk factor for complications, EEN might reduce the morbidity burden after PD.

4.2 Recruitment, screening and informed consent procedure

The study will be proposed to any patient planned for a PD meeting inclusion criteria. Of note, patients are usually referred to our consultation by external physicians or hospitals. The study will be presented to the patients during the first preoperative consultation by the investigators at the hospital. Expected benefits (fewer postoperative complications) and potential disadvantages as well as risks (poor tolerance of the nasojejunal tube, nausea, vomiting, diarrhea, tube obstruction, bronchial inhalation) will be explained. An information sheet will be given to the patient during the preoperative consultation. The patient will have the opportunity to ask questions.

A time of reflection will be given (at least 24 hours). The consent form will therefore be obtained at last the day before the intervention.

The investigators will explain to each participant the nature of the study, its purpose, the procedures involved, the expected duration, the potential risks and benefits and any discomfort it may entail. Each participant will be informed that the participation in the study is voluntary and that he or she may withdraw from the study at any time and that withdrawal of consent will not affect his or her subsequent medical assistance and treatment.

The participant will be informed that his or her medical records may be examined by authorized individuals other than their treating physician.

All participants for the study will be provided a participant information sheet and a consent form describing the study and providing sufficient information for participant to make an informed decision about their participation in the study.

The formal consent of a participant, using the approved consent form, will be obtained before the participant is submitted to any study procedure.

The consent form will be signed and dated by the investigator or his designee at the same time as the participant signs. A copy of the signed informed consent will be given to the study participant. The consent form will be retained as part of the study records. The informed consent process will be documented in the patient file and any discrepancy to the process described in this protocol will be explained.

4.3 Study procedures

Given the rate of annual procedures, a recruitment of about 60% of eligible patients, and based

on the experience of two randomized studies successfully completed in the Visceral Surgery Department (NCT0508300, NCT00512213), the planned overall study duration is three years including the recruitment period and follow-up. For patients the study duration will be from enrolment until POD 90, date of last follow-up phone call. The expected hospitalization duration for each patient will be approximately 14 days, which is the current mean hospital stay after PD in our department.

Eligibility of the patients will be confirmed on the day before the operation (day -1). Then, patients will be randomized before the operation (day -1) in either the control arm or the experimental one (EEN).

Standardized surgical procedure in all eligible patients: In terms of surgical details, all patients will receive a prophylactic dose of antibiotics (cefuroxime) 30 minutes before incision and first have exploratory laparotomy followed by conventional or pylorus-preserving PD. Pancreaticojejunostomy will be performed. End-to-side hepaticojejunostomy will be performed with single-layer interrupted sutures. A gastrojejunostomy will finally be constructed. Two perianastomotic drains will be placed.

Specific study procedure: At the end of surgery (after the three anastomoses are finished but before closure of the abdomen) and in the EEN study group only, a polyurethane single or double lumen feeding nasojejunal tube (Freka®) 8F will be inserted by the anesthesiologist and placed under direct palpation and visual control by the surgeon, 30 cm distally to the gastrojejunostomy into the jejunum. The tube will be attached according to current practice to the nose wing with a tape. The patient will therefore be under general anesthesia during tube insertion and no x-ray control will be needed.

Postoperatively, patients will receive standardized perioperative care according to the ERAS protocol in both arms.

From a nutritional point of view, this includes:

- The day before surgery: 2 carbohydrate drinks of 200 ml
- The operative day: 2 carbohydrate drinks of 200 ml up to 2 hours preoperatively, then postoperative free drinks
- On postoperative day (POD) 1: bouillons, creams, yogurts, drinks ≥2l
- On POD 2: light diet, drinks ≥2l
- On POD 3: normal diet (half portion)
- On POD 4: normal diet (full portion)

From POD 1, patients of both groups will receive two oral nutritional supplements (Resource® Ultra XS 125 ml, 280 kcal, 18 grams of proteins or analogous products) until discharge. In terms of intravenous infusions, a parenteral crystalloid solution will be used (Ringer-Lactate): 1000 ml during operative day and on POD 1, 500 ml during POD 2 and 3, then 250 ml, if necessary,

until POD 8 (minimum for maintenance of the central venous line). Anti-nausea agents (ondansetron 4 mg 3x/j and mephameson 4 mg 1x/j) as well as laxatives (magnesium hydroxyde 4.5 g 2x/j) will be used daily for 3 days, then on demand. Prokinetic agent (metoclopramide 10 mg 3x/j) will be used on demand. An anti-acid (esomeprazole 40 mg 1x/j) will be introduced for the duration of the hospitalization. Digestive enzymes will be prescribed from the first postoperative day (Creon 40'000 UI 3x/j).

In terms of mobilization, patients will be stimulated by nurses/physiotherapists according to the following plan:

- Operative day: just get up from bed
- On POD 1: walk once during the day, spend ≥6h out of bed (3 x 2h)
- On POD 2 to discharge: walk twice during the day, spend ≥ 8h out of bed (4 x 2h)

In our current practice, no suction gastric tube is left in place after the operation.

A standard nutrition protocol for the EEN intervention group will be prescribed as established in accordance with the nutritionists (see "3.4 Study intervention" for details). For patients randomized into the oral nutrition group they will receive the current postoperative management and receive from POD 1 an oral nutrition that will be gradually increased if tolerated until a normal diet (see above).

Several blood tests will be performed during the postoperative period. A timeline summary table of all study visits, relevant procedures, and samplings is shown in Appendix 1 (schedule of assessments).

Demographic disparities or difference in patient characteristics could be a source of bias. To reduce this risk, we decided to undertake a randomization of the participants. Moreover, heterogeneity in general management between centers might be a source of bias. Randomization will be stratified by center to decrease the risk of center bias.

4.4 Withdrawal and discontinuation

Patients will be withdrawn from the study if they leave the operation room with only a suction nasogastric tube in place or in case of withdrawal of informed consent, non-compliance to the study protocol, or due to safety concerns. Participants will not be replaced and considered as dropouts. Study data already collected on a participant until the time of withdrawal will still be used for analysis in a coded manner. No further data will be collected however from that time onwards.

5 STATISTICS AND METHODOLOGY

5.1. Statistical analysis plan and sample size calculation

A statistician was involved in the study design and estimate of the sample size.

Null hypothesis H0: EEN has no effect on postoperative complications (CCI) in the population (and therefore the observed effect is entirely due to chance): p2 = p1

Scientific hypothesis H1: EEN has an effect on postoperative morbidity (CCI) in the population (and therefore the observed effect is not entirely due to chance): p2 > p1

According to a previous randomized trial including a series of PD and assessing a realimentation process (enteral *vs.* parenteral), the mean CCI was impacted of about 30% (32.8 *vs.* 24.2)[24]. Another study reported a mean CCI of 38 after PD[38].

Based on the above results, we hypothesize that ENN will reduce by 30% a mean CCI of 35 (+/-20) of the oral nutrition group. We will therefore expect a mean CCI for the treatment group (EEN) of 24.5 (SD 20). In this superiority study, for a power of 80% and a significance level of p-value ≤0.05 (two-sided alpha), we will therefore need 57 patients per group according to the sample size calculation. Nevertheless, we will increase the sample size to a total of 144 patients to take into account 20% of drop-outs (e.g., due to non realization of pancreatoduodenectomy or consent withdraw) at 90 days (primary endpoint evaluation). We will therefore need to enroll 72 patients per group in the trial.

The study will be closed once the required 144 patients will be included. No interim analysis will be performed.
Normality of distribution will be determined by the Kolmogorov-Smirnov test and quantile-quantile plots of dependent variables for all continuous variables.

We will use a Student *t*-test to evaluate if the primary outcome (CCI) can significantly be reduced by EEN compared to the control group (comparison of mean CCI hypothesizing a normal distribution). For the analysis of all secondary endpoints, we will also use *t*-tests or chi-squared tests based on the variable types. Regarding the questionnaires filled 4 times during the study, tests specific to repetitive ordinal measures will be used.

The analysis will be based on the intention-to-treat method and not per protocol. We will perform an intention-to-treat analysis so that all patients being intended to treat will be analyzed in the statistics independently. All patients will therefore be analyzed according to the group in which they were initially randomized.

Blocked randomization will be done using a computerized algorithm via REDCap by a research coordinator (different from the person who included the patient) the day before surgery. The proportion of "study" (EEN) and "control" (oral nutrition) subjects will be 1:1 (mix of variable block sizes of 4, 6, and 8 patients, randomly selected). Before surgery, only the responsible surgeon will know the allocation group. Postoperatively, the inclusion in the different groups will be known by the caregiver team and the patient, as it is not possible to blind the intervention (nasojejunal tube).

The investigators, the outcome adjudicators, and the data analysts will be blinded (allocation concealment).

The statistical package used for analysis will be SPSS version 26 (IBM Corp., Armonk, NY, USA).

5.2. Handling of missing data and drop-outs

In case of missing data among variables other than endpoints (adjustment variables, >5% of expected data) we will consider the use of the multiple imputation technique. This process will be performed multiple times (e.g., 10-20 times) to combine multiple data sets to produce one final data sets replacing the missing data[39].

A 20% drop-out was considered in the sample size calculation.

6 REGULATORY ASPECTS AND SAFETY

6.1 Local regulations / Declaration of Helsinki

This study is conducted in compliance with the protocol, the current version of the Declaration of Helsinki, the ICH-GCP, the HRA as well as other locally relevant legal and regulatory requirements.

6.2 Serious Adverse Events and notification of safety and protective measures

An Adverse Event (AE) is any untoward medical occurrence in a patient or a clinical investigation subject which does not necessarily have a causal relationship with the trial procedure. An AE can therefore be any unfavourable or unintended finding, symptom, or disease temporally associated with a trial procedure, whether or not related to it.

A Serious Adverse Event (SAE) (ClinO, Art. 63) is any untoward medical occurrence that

- Results in death or is life-threatening,
- Requires in-patient hospitalization or prolongation of existing hospitalization,
- Results in persistent or significant disability or incapacity, or
- Causes a congenital anomaly or birth defect

Both Investigator and Sponsor make a causality assessment of the event to the trial intervention, (see table below based on the terms given in ICH E2A guidelines). Any event assessed as possibly, probably or definitely related is classified as related to the trial intervention.

Relationship	Description
Definitely	Temporal relationship<br>Improvement after dechallenge*<br>Recurrence after rechallenge<br>(or other proof of drug cause)
Probably	Temporal relationship<br>Improvement after dechallenge<br>No other cause evident
Possibly	Temporal relationship<br>Other cause possible
Unlikely	Any assessable reaction that does not fulfil the above conditions
Not related	Causal relationship can be ruled out
*Improvement after dechallenge only taken into consideration, if applicable to reaction	

Investigators make a severity assessment of the event as mild, moderate or severe. Mild means the complication is tolerable, moderate means it interferes with daily activities and severe means it renders daily activities impossible.

Reporting of SAEs (see ClinO, Art. 63)
All SAEs are documented and reported immediately (within a maximum of 24 hours) to the Sponsor of the study.

If it cannot be excluded that the SAE occurring in Switzerland is attributable to the intervention under investigation (related to enteral nutrition), the Investigator reports it to the Ethics Committee via BASEC within 15 days.

If the SAE occurs at one of the study sites, the coordinating Investigator reports the events to the Ethics Committee concerned, within 15 days.

Exemptions from expedited reporting may be possible if the SAE is a clear complication of the operation (pancreatoduodenectomy) not related to the study intervention (enteral nutrition) or if the SAE corresponds to a rehospitalization or prolongation of the hospitalization due to a postoperative complication unlinked to the study intervention (enteral nutrition).

Follow up of (Serious) Adverse Events
Participants who experience adverse events will be retained in the study. A follow-up of SAE will be organized accordingly, under the supervision of the sponsor, depending on the type of event. Patients with SAE will be followed until resolution or stabilization of the effect will be noted.

Notification of safety and protective measures (see ClinO, Art 62, b)

If immediate safety and protective measures have to be taken during the conduct of the study, the investigator notifies the Ethics committee of these measures, and of the circumstances

necessitating them, within 7 days.

6.3 (Periodic) safety reporting

An annual safety report (ASR/DSUR) is submitted once a year to the local Ethics Committee by the Investigator (ClinO, Art. 43 Abs).

In international multicentric studies the ASR/DSUR contains information from all sites including information from sites outside of Switzerland. The Sponsor distributes the ASR/DSUR to all the participating Investigators.

6.4 Radiation

N/A.

6.5 Amendments

Substantial changes to the study setup and study organization, the protocol and relevant study documents are submitted to the Ethics Committee for approval before implementation. Under emergency circumstances, deviations from the protocol to protect the rights, safety and well-being of human subjects may proceed without prior approval of the Ethics Committee. Such deviations shall be documented and reported to the Ethics Committee as soon as possible.

A list of all non-substantial amendments will be submitted once a year to the competent EC together with the ASR.

6.6 (Premature) termination of study

The Sponsor may terminate the study prematurely according to certain circumstances, e.g.

- Ethical concerns,
- Insufficient participant recruitment,
- When the safety of the participants is doubtful or at risk (e.g. when the benefit-risk assessment is no longer positive),
- Alterations in accepted clinical practice that make the continuation of the study unwise, or
- Early evidence of harm or benefit of the experimental intervention

Upon regular study termination, the Ethics Committee is notified via BASEC within 90 days (ClinO, Art. 38).

Upon premature study termination or study interruption, the Ethics Committee is notified via BASEC within 15 days (ClinO, Art. 38).

Health-related data at the end of the study will be coded upon end of data analysis.

6.7 Insurance

In the event of study-related damage or injuries, the liability of the institution CHUV (centre hospitalier universitaire vaudois) provides compensation, except for claims that arise from misconduct or gross negligence.

7 FURTHER ASPECTS

7.1 Overall ethical considerations

The study design (randomized controlled trial) will permit to have a good internal validity of the study. Moreover, the CCI used as main outcome is a validated index of general postoperative morbidity and it enables to encompass all complications that a patient may present. The inclusion of several centers internationally will increase the generalizability of the results (external validity). The complication rate after PD remains high (around 60%) and malnutrition has been established as a risk factor of postoperative complication. An intervention that could improve the nutritional status of the patients might lead to a decrease of morbidity after PD.

If the results are favorable, this study will permit to establish an EEN protocol to improve patient outcomes after PD. Patients undergoing PD could rapidly benefit of this management, and EEN could become the new standard of care for the perioperative nutrition management after PD. The results of this study could be implemented and translated into daily clinical practice promptly.

The need for research in this field is clearly present, as the issue of postoperative nutrition after PD is not resolved and does not reach an overall consensus among international pancreatic surgeons. The numerous presentations, debates in congresses, and our recently published survey[40] on that subject attest and highlight the absence of universal consensus and lack of solid data.

The results of this study would go beyond the only scientific interest, as they will directly impact patients undergoing pancreas surgery. As pancreas cancer incidence is projected to grow in the upcoming years, pancreas surgery number will correlatively increase. Ultimately, in the current era of growing health expenditures and need for cost containment, if EEN allows decreasing complications and length of stay, it could also decrease the overall costs for each patient hospitalization for PD, which could have important positive repercussions on the health care system.

Particular attention will be paid to the process of randomization in order to ensure a sound methodology.

An overall fair balance for the study participant will be maintained.

7.2 Risk-benefit assessment

There are several adverse events associated with nasojejunal tube and enteral nutrition: poor

tolerance, nausea, vomiting, diarrhea, tube obstruction, or bronchial inhalation. Nursing teams are trained to use the equipment and will perform the same care as usual: position verification, flushing, nasal fixation, nasal eschar surveillance. With these measures, the risk of adverse events associated with the study is judged to be low.

From the investigators‘ perspective, we hypothesize that the EEN intervention will be a benefit for patients included in the study group. Benefits of EEN could be a decrease of postoperative complications and a shorter length of stay. Nevertheless, the control group cannot be considered disadvantaged as currently no intervention of this type exists in visceral surgery. It is also possible that the participation to the study will not bring any benefits.

8 QUALITY CONTROL AND DATA PROTECTION

8.1 Quality measures

A research nurse or collaborator will be hired for this study. He/she will have specific training in all important study-related aspects. Monitoring of the study will be performed by the clinical research center (CRC) of the CHUV.

For quality assurance the sponsor, the Ethics Committee or an independent trial monitor may visit the research sites. Direct access to the source data and all study related files is granted on such occasions. All involved parties keep the participant data strictly confidential.

8.2 Data recording and source data

Study data will be recorded using electronic Case Report Form created on REDCap. This software contains among others an audit trail. Each participant will have an individual CRF. CRF will not identify patients by their name or birth date but will have a coded identification (e.g., patient 1: 23, patient 2: 14, or patient 3: 46).

The source data of this study are the original medical records of the patients containing clinical, biological, and radiological findings. All data will be recorded using eCRF documents. Standardized questionnaires (EORTC) will also be distributed to the patients and compiled.

8.3 Confidentiality and coding

Trial and participant data will be handled with uttermost discretion and are only accessible to authorized personnel who require the data to fulfil their duties within the scope of the study. On the eCRFs and other study specific documents, participants are only identified by a unique participant number.

A research coordinator of our division will store in his/her office the identification list in a key-closed drawer.

Audit trail embedded in the database (REDCap) will ensure traceability according to ClinO, Art. 18. Only password-protected access will be permitted and safety backups on storage media (CHUV secure servers) will prevent misuse of recorded information.

Only non-genetic data will be used.

8.4 Retention and destruction of study data and biological material

Appropriate data archiving is the responsibility of the PI of the study. All study data are archived for 10 years after study termination or premature termination of the study in a coded manner. All the study data collected will be archived on a CHUV network storage server whose integrity, security and backups are provided by the department of informatic system (DSI).

No biological material will be conserved specifically for this study.

9 MONITORING AND REGISTRATION

Monitoring activities have the purpose to ensure that the study is conducted in accordance with the clinical study protocol, applicable laws, regulations and procedures. This includes verifying the quality and integrity of data and the protection of the rights, welfare, and safety of subjects participating in the study. The CRC of the CHUV will monitor the study for the centers in Switzerland. The CRC appointed monitor will be responsible for ensuring that the activities are conducted in compliance with the ICH-GCP E6 (R2) guidelines, the Declaration of Helsinki, all applicable sponsor requirements for the study in object as well as any applicable local law and regulation. It will be the responsibility of the sponsor to ensure that all monitor working in the context of the study is adequately trained and complies to all applicable sponsor requirements. The necessary visits for the monitoring will be done following the recommendations of the monitoring committee of the CRC. Visits prior the start, in the middle, and at the end of the study will be realized. Data issued from the eCRF and the database will be monitored. The same monitoring will be applied by the centers outside of Switzerland.

The source data and documents will be accessible to monitors and potential questions will be answered during monitoring.

The present study will be registered in the Swiss National Clinical trial Portal (SNCTP via BASEC). Moreover, this trial will be registered in ClinicalTrials.gov and the protocol of this study will be published in a peer-review journal such as *Trials* or *BMJ Open*.

10 FUNDING / PUBLICATION / DECLARATION OF INTEREST

Fundings of CHF 20’000 and CHF 100’000 were received from the Livio-Glauser Foundation and from the Valery Foundation for this study. Moreover, the department of Visceral Surgery will also

provide some funding.

This study should enable to publish several manuscripts in peer-reviewed journals with high impact in the surgical community. Preparation of the articles will start as soon as inclusion of all patients will be closed. The investigators declare to have no conflict of interest to disclose.

11 REFERENCES

1. Siegel RL, Miller KD, Jemal A. Cancer statistics, 2020. CA Cancer J Clin. 2020;70(1):7–30.

2. Rahib L, Smith BD, Aizenberg R, Rosenzweig AB, Fleshman JM, Matrisian LM. Projecting cancer incidence and deaths to 2030: the unexpected burden of thyroid, liver, and pancreas cancers in the United States. Cancer Res. 2014 Jun 1;74(11):2913–21.

3. Fristrup C, Detlefsen S, Hansen CP, Ladekarl M. Danish Pancreatic Cancer Database. Clin Epidemiol. 2016;8:645–8.

4. Hartwig W, Hackert T, Hinz U, Gluth A, Bergmann F, Strobel O, et al. Pancreatic cancer surgery in the new millennium: better prediction of outcome. Ann Surg. 2011 Aug;254(2):311–9.

5. Simons JP, Shah SA, Ng SC, Whalen GF, Tseng JF. National complication rates after pancreatectomy: beyond mere mortality. J Gastrointest Surg. 2009 Oct;13(10):1798–805.

6. Winter JM, Cameron JL, Campbell KA, Arnold MA, Chang DC, Coleman J, et al. 1423 pancreaticoduodenectomies for pancreatic cancer: A single-institution experience. J Gastrointest Surg. 2006 Nov;10(9):1199–210; discussion 1210-1211.

7. Bozzetti F, Mariani L. Perioperative nutritional support of patients undergoing pancreatic surgery in the age of ERAS. Nutrition. 2014 Dec;30(11–12):1267–71.

8. Bozzetti F, Gianotti L, Braga M, Di Carlo V, Mariani L. Postoperative complications in gastrointestinal cancer patients: the joint role of the nutritional status and the nutritional support. Clin Nutr. 2007 Dec;26(6):698–709.

9. Schiesser M, Müller S, Kirchhoff P, Breitenstein S, Schäfer M, Clavien P-A. Assessment of a novel screening score for nutritional risk in predicting complications in gastro-intestinal surgery. Clin Nutr. 2008 Aug;27(4):565–70.

10. Sungurtekin H, Sungurtekin U, Balci C, Zencir M, Erdem E. The influence of nutritional status on complications after major intraabdominal surgery. J Am Coll Nutr. 2004 Jun;23(3):227–32.

11. Mazaki T, Ebisawa K. Enteral versus parenteral nutrition after gastrointestinal surgery: a systematic review and meta-analysis of randomized controlled trials in the English literature. J Gastrointest Surg. 2008 Apr;12(4):739–55.

12. Braunschweig CL, Levy P, Sheean PM, Wang X. Enteral compared with parenteral nutrition: a meta-analysis. Am J Clin Nutr. 2001 Oct;74(4):534–42.

13. Braga M, Gianotti L, Gentilini O, Parisi V, Salis C, Di Carlo V. Early postoperative enteral

nutrition improves gut oxygenation and reduces costs compared with total parenteral nutrition. Crit Care Med. 2001 Feb;29(2):242–8.

14. Bozzetti F, Braga M, Gianotti L, Gavazzi C, Mariani L. Postoperative enteral versus parenteral nutrition in malnourished patients with gastrointestinal cancer: a randomised multicentre trial. Lancet. 2001 Nov 3;358(9292):1487–92.

15. Baradi H, Walsh RM, Henderson JM, Vogt D, Popovich M. Postoperative jejunal feeding and outcome of pancreaticoduodenectomy. J Gastrointest Surg. 2004 Jun;8(4):428–33.

16. Slankamenac K, Graf R, Barkun J, Puhan MA, Clavien P-A. The comprehensive complication index: a novel continuous scale to measure surgical morbidity. Ann Surg. 2013 Jul;258(1):1–7.

17. Dindo D, Demartines N, Clavien P-A. Classification of surgical complications: a new proposal with evaluation in a cohort of 6336 patients and results of a survey. Ann Surg. 2004 Aug;240(2):205–13.

18. Horan TC, Gaynes RP, Martone WJ, Jarvis WR, Emori TG. CDC definitions of nosocomial surgical site infections, 1992: a modification of CDC definitions of surgical wound infections. Infect Control Hosp Epidemiol. 1992 Oct;13(10):606–8.

19. Bassi C, Dervenis C, Butturini G, Fingerhut A, Yeo C, Izbicki J, et al. Postoperative pancreatic fistula: an international study group (ISGPF) definition. Surgery. 2005 Jul;138(1):8–13.

20. Wente MN, Bassi C, Dervenis C, Fingerhut A, Gouma DJ, Izbicki JR, et al. Delayed gastric emptying (DGE) after pancreatic surgery: a suggested definition by the International Study Group of Pancreatic Surgery (ISGPS). Surgery. 2007 Nov;142(5):761–8.

21. Wente MN, Veit JA, Bassi C, Dervenis C, Fingerhut A, Gouma DJ, et al. Postpancreatectomy hemorrhage (PPH): an International Study Group of Pancreatic Surgery (ISGPS) definition. Surgery. 2007 Jul;142(1):20–5.

22. Specimen-QLQ-C30-English.pdf [Internet]. [cited 2020 Oct 28]. Available from: https://www.eortc.org/app/uploads/sites/2/2018/08/Specimen-QLQ-C30-English.pdf

23. Kondrup J, Allison SP, Elia M, Vellas B, Plauth M, Educational and Clinical Practice Committee, European Society of Parenteral and Enteral Nutrition (ESPEN). ESPEN guidelines for nutrition screening 2002. Clin Nutr. 2003 Aug;22(4):415–21.

24. Perinel J, Mariette C, Dousset B, Sielezneff I, Gainant A, Mabrut J-Y, et al. Early Enteral Versus Total Parenteral Nutrition in Patients Undergoing Pancreaticoduodenectomy: A Randomized Multicenter Controlled Trial (Nutri-DPC). Ann Surg. 2016 Nov;264(5):731–7.

25. Melloul E, Lassen K, Roulin D, Grass F, Perinel J, Adham M, et al. Guidelines for Perioperative Care for Pancreatoduodenectomy: Enhanced Recovery After Surgery (ERAS) Recommendations 2019. World J Surg. 2020 Jul;44(7):2056-84.

26. Gudjonsson B. Pancreatic Cancer: 80 Years of Surgery-Percentage and Repetitions. HPB

Surg. 2016;2016:6839687.

27. Greco M, Capretti G, Beretta L, Gemma M, Pecorelli N, Braga M. Enhanced recovery program in colorectal surgery: a meta-analysis of randomized controlled trials. World J Surg. 2014 Jun;38(6):1531–41.

28. Coolsen MME, van Dam RM, van der Wilt AA, Slim K, Lassen K, Dejong CHC. Systematic review and meta-analysis of enhanced recovery after pancreatic surgery with particular emphasis on pancreaticoduodenectomies. World J Surg. 2013 Aug;37(8):1909–18.

29. Joliat G-R, Labgaa I, Petermann D, Hübner M, Griesser A-C, Demartines N, et al. Cost-benefit analysis of an enhanced recovery protocol for pancreaticoduodenectomy. Br J Surg. 2015 Dec;102(13):1676–83.

30. Joliat G-R, Ljungqvist O, Wasylak T, Peters O, Demartines N. Beyond surgery: clinical and economic impact of Enhanced Recovery After Surgery programs. BMC Health Serv Res. 2018 Dec 29;18(1):1008.

31. Joliat G-R, Hübner M, Roulin D, Demartines N. Cost Analysis of Enhanced Recovery Programs in Colorectal, Pancreatic, and Hepatic Surgery: A Systematic Review. World J Surg. 2020 Mar;44(3):647–55.

32. Morera-Ocon FJ, Sabater-Orti L, Muñoz-Forner E, Pérez-Griera J, Ortega-Serrano J. Considerations on pancreatic exocrine function after pancreaticoduodenectomy. World J Gastrointest Oncol. 2014 Sep 15;6(9):325–9.

33. Cederholm T, Bosaeus I, Barazzoni R, Bauer J, Van Gossum A, Klek S, et al. Diagnostic criteria for malnutrition - An ESPEN Consensus Statement. Clin Nutr. 2015 Jun;34(3):335–40.

34. Sorensen J, Kondrup J, Prokopowicz J, Schiesser M, Krähenbühl L, Meier R, et al. EuroOOPS: an international, multicentre study to implement nutritional risk screening and evaluate clinical outcome. Clin Nutr. 2008 Jun;27(3):340–9.

35. Gerritsen A, Besselink MG, Cieslak KP, Vriens MR, Steenhagen E, van Hillegersberg R, et al. Efficacy and complications of nasojejunal, jejunostomy and parenteral feeding after pancreaticoduodenectomy. J Gastrointest Surg. 2012 Jun;16(6):1144–51.

36. Gerritsen A, Besselink MGH, Gouma DJ, Steenhagen E, Borel Rinkes IHM, Molenaar IQ. Systematic review of five feeding routes after pancreatoduodenectomy. Br J Surg. 2013 Apr;100(5):589–98; discussion 599.

37. Quality of Life of Cancer Patients [Internet]. EORTC – Quality of Life. [cited 2020 Mar 26]. Available from: https://qol.eortc.org/questionnaire/eortc-qlq-c30/

38. Abou Khalil J, Mayo N, Dumitra S, Jamal M, Chaudhury P, Metrakos P, et al. Pancreatic fistulae after a pancreatico-duodenectomy: are pancreatico-gastrostomies safer than pancreatico-jejunostomies? An expertise-based trial and propensity-score adjusted analysis. HPB (Oxford). 2014 Dec;16(12):1062–7.

39. Rubin DB, Schenker N. Multiple imputation in health-care databases: an overview and

some applications. Stat Med. 1991 Apr;10(4):585–98.

40. Martin D, Joliat G-R, Halkic N, Demartines N, Schäfer M. Perioperative nutritional management of patients undergoing pancreatoduodenectomy: an international survey among surgeons. HPB (Oxford). 2020 Jan;22(1):75–82.

Appendix 1: Schedule of assessments

Study periods	Screening	Entry	Intervention			Discharge	Follow-up	
Visits	1	2	Daily			3	4	5
Days	Preoperative	-1	0	1-7	Hospitalization	14^	30-45	90
Patient information and informed consent	x*	x*						
Patient eligibility confirmation		x						
Demographics	x	x						
Randomization		x						
Standard surgery (not study procedure)			x					
Nasojejunal tube (EEN group)			x***					
Physical examination	x	x	Daily			x	x	
Vital signs	x	x	Daily			x	x	
Metabolic tests ¥		x			2x/week			
Nutrition tests Ω		x					x	
Body measures°		x				x	x	
Indirect calorimetry				on POD 5				
EORTC QLQ-C30	x**	x**				x	x	x
Subjective tolerance (VAS 0-10)				x				
Complications - CCI						x	x	x
Complications - Clavien						x	x	x
LOS						x		
Readmissions								x

Demographics include the measure of serum CA 19-9 at admission in case of pancreatic cancer.

EEN: early enteral nutrition, **EORTC**: European Organisation for Research and Treatment of Cancer, **VAS:** Visual Analog Scale, **CCI**: Comprehensive Complication Index, **LOS**: length of stay, **POD:** postoperative day.

¥ **Metabolic tests**: CRP, procalcitonin, simple blood count, coagulation tests, electrolytes (sodium, potassium, calcium, magnesium, phosphate), creatinine, urea, blood glucose, liver and pancreatic function tests, prealbumin, albumin, triglycerides.

Ω **Nutrition tests:** folate, magnesium, iron, ferritin, transferrin saturation, vitamin D, calcium, phosphate and parathormone, vitamin E, HbA1c (glycated hemoglobin).

° **Body measures:** lean body mass using bioelectrical impedance analysis and strength using handgrip.

* Filled either during screening visit or at hospital admission.

** The preoperative EORTC questionnaire will be filled during screening visit or on hospital entry day. In total, four questionnaires will be filled. The last one on postoperative day 90 will be filled by the study nurse who will perform the phone call.

*** At the end of surgery and in the EEN study group only: polyurethane single or double lumen feeding nasojejunal tube (Freka®) 8F.

^ Discharge on day 14: current mean hospital stay.

A maximum of six months is permitted between the screening and the intervention. If the interval is longer, a new (more recent) consent form will be signed.

The calorimetry should ideally be performed between POD 3 and POD 7. The accepted period to perform the calorimetry taking into account the clinical variability is between POD 3 and POD 30.

The visit n°4 should ideally be performed between POD 30 and POD 45. The accepted period to do the visit n°4 taking into account the clinical variability is between POD 20 and POD 60.

The visit n°5 should ideally be performed on POD 90. The accepted period to do the visit n°5 taking into account the clinical variability is between POD 75 and POD 115.

The most important questionnaire is the questionnaire of the visit n°5 on POD 90. If this questionnaire is not performed, it will be considered a deviation to the protocol. Absence of other questionnaires will not be considered as protocol deviation.
