# Supplementary material for: Early Enteral vs Oral Postoperative Nutrition After Pancreatoduodenectomy: The NUTRIWHI Randomized Clinical Trial
Source: JAMA Surg. 2026 Apr 22;161(6):575–82. doi: 10.1001/jamasurg.2026.1048 (PMC13103875; doi:10.1001/jamasurg.2026.1048)
Supplement: Supplement 2. — eMethods Enteral and oral nutrition protocols Perioperative management Study protocol and role of the funding sources Definitions Statistical analyses eTable 1. Comparisons between excluded and included patients eResults. Sensitivity analyses eFigure 1. Subgroup analyses and Kaplan-Meier graph eTable 2. Complementary analyses on main outcome eReferences [file jamasurg-e261048-s002.pdf]

Supplemental Online Content

Joliat GR, Martin D, Labgaa I, et al. Early enteral vs oral postoperative nutrition after pancreatoduodenectomy: the NUTRIWHI randomized clinical trial. *JAMA Surg*. Published online April 22. 2026. doi:10.1001/jamasurg.2026.1048

eMethods
Enteral and oral nutrition protocols
Perioperative management
Study protocol and role of the funding sources
Definitions
Statistical analyses
eTable 1. Comparisons between excluded and included patients
eResults.
Sensitivity analyses
Subgroup analyses and Kaplan-Meier graph (eFigure 1)
Complementary analyses on main outcome (eTable 2)
eReferences.

This supplemental material has been provided by the authors to give readers additional information about their work.

© 2026 Joliat et al. *JAMA Surg*.

eMethods. Enteral and oral nutrition protocols

Enteral nutrition
Enteral nutrition was given in the enteral nutrition group following this protocol:

- Six hours after the operation: low flow enteral feeding with Isosource® Energy Fibre solution (or similar product, 400 kcal) at a speed of 21 ml/h (250 ml/12h).
- If tolerance was subjectively good (visual analogue scale ≤4/10), increased flow to 42 ml/h (500 ml/12h from 8 pm to 8 am, 800 kcal) on postoperative day (POD) 1.
- On POD 2: increased flow to 62.5 ml/h (750 ml/12h, 1200 kcal)
- On POD 3: increased flow to 83.5 ml/h (1000 ml/12h, 1600 kcal)

If the tolerance was not satisfactory (5/10 and 6/10), the current flow was maintained 24 hours more. It was decreased to previous stage if tolerance was >6/10 or put on hold for 6 hours in case of persisting digestive symptoms (severe nausea, vomiting, severe bloating or severe diarrhea) despite diminution of the nutrition flow, and increased the next day if tolerated until the maximum of 1000 ml/12h.

The diet was infused over 12 hours with a pump and controlled flow rate. Enteral nutrition was stopped when oral food intake reached more than 50% of nutritional requirements.

Oral nutrition
Regarding oral diet in both groups, the protocol was the following:

- Day before surgery: 2 carbohydrate drinks of 200 ml
- Operative day: 2 carbohydrate drinks of 200 ml up to 2 hours preoperatively, then postoperative free drinks
- On POD 1: broths, creams, yogurts, drinks ≥2l
- On POD 2: light diet, drinks ≥2l
- On POD 3: normal diet (half serving)
- On POD 4: normal diet (full serving)

From POD 1, patients of both groups received two oral nutritional supplements (Resource® Ultra XS 125 ml, 280 kcal, 18 grams of proteins or analogous products) until discharge. Digestive enzymes (Creon 40’000 UI 3 times a day) were prescribed from POD 1.

Perioperative management

Patients received prophylaxis antibiotics before incision with cefuroxime. Metronidazole was added if patients had a preoperative biliary drainage. No routine postoperative prophylaxis antibiotics were given unless patients had preoperative biliary drainage. In that case, piperacillin-tazobactam was given for 5 days. Patients followed an enhanced recovery pathway postoperatively (already described in detail in previous articles by our group)[1,2] with strong emphasis on mobilization and respiratory physiotherapy. Drain was removed if amylase level in the drain fluid was lower than 3 times blood values on postoperative day 5.

Study protocol and role of the funding sources

The study protocol was published in open access and is freely available online (https://www.frontiersin.org/journals/oncology/articles/10.3389/fonc.2022.855784/full).
The Livio-Glauser foundation and the Valery foundation did not play a role in the study design, data collection, analysis, interpretation of data, writing of the report, and decision to submit the paper for publication.

Definitions

© 2026 Joliat et al. *JAMA Surg*.

The nutritional status of the nutritional risk screening (NRS) is based on the weight loss, the body mass index (BMI), and the food intake (range 0-3). The severity of the disease of the NRS also ranges from 0 to 3. The final NRS is calculated by adding the nutritional status score and the score of the disease severity, and one point is added if the patient age is ≥70 years.

A score ≥3 indicates a patient at nutritional risk and this threshold was taken as an inclusion criterion. Length of stay was calculated in days from operation day to hospital discharge.
Adverse events were defined in the enteral group as untoward occurrences associated with the enteral nutrition (such as abdominal pain during enteral nutrition) or the nasojejunal tube (e.g., involuntarily tube removal or tube obstruction). Enteral nutrition tolerance was assessed daily using a 0-10 scale (0=no symptom, 10=major discomfort).

Statistical analyses

Of note, the initial drop-out rate was set at 10% and the estimated sample size was 128 patients (as it appears on the published study protocol). Following the inclusion of the first 50 patients, as the drop-out rate was >10%, the study sample size was increased to 144 (based on a 20% drop-out rate) after granting approval by the local ethics committee.
The subgroup analyses were not pre-specified in the protocol and were post hoc analyses. Interaction tests were calculated for each subgroup analysis. Moreover, to account for multiple comparisons, a significant threshold was defined as 1% using the Bonferroni correction formula (as 5 comparisons were performed: 0.05/5=0.01). Missing data were specified, if necessary, in the tables of the results.
A Kaplan-Meier analysis was used to depict the morbidity plotted with the length of stay (eFigure 1). Events were defined as occurrence of a postoperative complication within 90 days, and patients were censored at the end of the 90-day follow-up or if dead before postoperative day 90. Comparison between enteral and oral groups was made using log-rank test.
The study was closely monitored by an independent monitoring team from the clinical research unit of the Lausanne University Hospital. In addition to implementation and closing monitoring visits, 6 intermediate visits were performed to ensure the study followed the protocol and the regulations.

Comparisons between excluded and included patients

eTable 1. Preoperative characteristics of excluded (dropout after randomization) and included patients

	Excluded patients n=24	Included patients n=118	Mean difference (95% CI) or RR (95% CI)	p-value
Age, years	69 (61-74)	70 (62-76)	0.5 (-3.2 to 4.3)	0.787
Women/men (%)	13 (54%)/11 (46%)	51 (43%)/67 (57%)	1.1 (0.7-1.7)	0.542
BMI, kg/m^2	24 (21-27)	25 (22-28)	0.8 (-1.1 to 2.7)	0.384
Neoadjuvant treatment (%)	5 (21%)	29 (25%)	0.9 (0.7-1.1)	0.403

© 2026 Joliat et al. *JAMA Surg*.

Diabetes (%)	8 (33%)	26 (22%)	1.2 (0.9-1.6)	0.237
Active smoking (%)	9 (38%)	24 (20%)	1.3 (0.9-1.8)	0.052
Jaundice (clinical diagnosis before biliary drainage if performed) (%)	15 (63%)	72 (61%)	1.1 (0.6-1.9)	0.831
Preoperative biliary drainage (%)	14 (58%)	69 (58%)	1.0 (0.6-1.7)	0.949
Nutritional risk screening, Kondrup score (%)<br>3<br>4/5/6	 <br>7 (29%)<br>17 (71%)	 <br>54 (46%)<br>64 (54%)	0.8 (0.6-1.1)	0.134
ASA score (%)<br>I/II<br>III/IV	 12 (50%)<br>12 (50%)	 73 (62%)<br>45 (38%)	0.8 (0.5-1.2)	0.280
Day before surgery prealbumin value, g/l	0.21 (0.18-0.28)	0.22 (0.19-0.26)	-0.01 (-0.03 to 0.03)	0.938
Highest preoperative CA19-9 during the month prior the operation, U/ml	71 (18-669)	58 (9-482)	-8142 (-29859 to 13575)	0.413
Etiologies (%)<br>- Malignant<br>- Non malignant	 20 (83%)<br>4 (17%)	 93 (79%)<br>25 (21%)	1.3 (0.5-3.3)	0.617

Continuous variables are presented with median and interquartile range, and categorial variables with number and percentage.
BMI: body mass index, ASA: American Society of Anesthesiologists, CA: carbohydrate antigen, PDAC: pancreatic ductal adenocarcinoma, PNET: pancreatic neuroendocrine tumor, IPMN: intraductal papillary and mucinous neoplasm, CI: confidence interval, RR: risk ratio.

© 2026 Joliat et al. *JAMA Surg*.

eResults

Sensitivity analyses

Per protocol analysis
Three patients allocated in the enteral group did not receive enteral nutrition during their hospital stay. For the per protocol analysis, they were therefore counted in the oral group, leaving 56 patients in the enteral group and 62 in the oral group. Mean CCI was 24.7 ± 21.3 in the enteral group and 36.1 ± 24.7 in the oral group (mean difference 11.4, 95% CI 2.9-19.8, p=0.009).

Morbidity analysis without perioperative deaths
Two patients died during the 90-day postoperative period (one in each group). If these 2 patients are excluded from the analysis, therefore comparing 58 patients in the enteral group and 58 patients in the oral group, mean CCI was significantly lower in the enteral group (24.2 ± 18.9 *vs*. 34.7 ± 24.1, mean difference 10.5, 95% CI 2.9-18.8, p=0.008).

Patients without need of parenteral nutrition
Twenty-nine patients in the enteral group and 27 patients in the oral group did not need supplemental parenteral nutrition. In this subgroup, the mean CCI was lower in the enteral group than in the oral group (13.9 ± 20.5 *vs*. 27.2 ± 22.6, mean difference 13.4, 95% CI 1.9-24.9, p=0.024)

Subgroup analyses and Kaplan-Meier graph (eFigure 1)

Interaction tests for the primary subgroup analysis shown in the article
To test potential interactions between the variables included in the post hoc subgroup analysis (age, diabetes, body mass index, preoperative biliary drainage, and NRS), interaction terms were created and were tested in a multiple linear regression model. Unstandardized coefficients, 95% CI, and p-values for each interaction term are shown hereunder:
Age and diabetes: -0.4, 95% CI -1.4 to 0.5, p=0.373
Age and body mass index: -0.002, 95% CI -0.1 to 0.1, p=0.964
Age and preoperative biliary drainage: -0.7, 95% CI -1.6 to 0.2 , p=0.141
Age and NRS: 0.2, 95% CI -0.2 to 0.6, p=0.369
Diabetes and body mass index: 0.2, 95% CI -2.1 to 2.5, p=0.854
Diabetes and preoperative biliary drainage: -9.9, 95% CI -31.0 to 11.2, p=0.353
Diabetes and NRS: 7.6, 95% CI -4.4 to 19.7, p=0.213
Body mass index and preoperative biliary drainage: -1.6, 95% CI -3.4 to 0.2, p=0.078
Body mass index and NRS: -0.4, 95% CI -1.3 to 0.6, p=0.417
Preoperative biliary drainage and NRS: 7.2, 95% CI -2.8 to 17.2, p=0.157

Analyses by center
After inclusion, ninety-eight patients were from center A and 20 patients were from center B and C. The results for the mean CCI in each center were as follows:

- Center A (n=98): mean CCI, supplemental enteral group: 28.3 ± 17.7 *vs*. oral group: 38.6 ± 24.6, mean difference -10.4, 95% CI -19.0 to -1.8 , p=0.018
- Center B/C (n=20): mean CCI, supplemental enteral group: 12.1 ± 9.9 *vs*. oral group: 22.1 ± 24.3, mean difference -10.0, 95% CI -36.2 to 16.3, p=0.435

Even though Center A has means in both groups that are higher than in Center B/C, the mean differences in Center A (-10.4) and Center B/C (-10.0) have approximately the same magnitude. The larger sample size in Center A is largely responsible for the difference in p-values. It should be noted that the present study may not be well powered to detect a center effect.

Patients with a nutritional risk screening (NRS) ≥5
In the present cohort, 27 patients had a NRS ≥5 (14 in the oral group and 13 in the enteral group). The mean CCI was lower in the enteral group than in the oral group (24.7 ± 14.1 *vs*. 42.3 ± 24.2, mean difference -17.5, 95 % CI -34.2 to -0.9, p=0.040).

Patients with pancreatic ductal adenocarcinoma

© 2026 Joliat et al. *JAMA Surg*.

Twenty-eight patients with PDAC were included in the supplemental enteral group and 27 in the oral alone group. The mean CCI for the supplemental enteral group was 17.1 ± 17.7 and 30.2 ± 23.4 for the oral group (mean difference -13.2, 95% CI -24.7 to -1.6, p=0.026).

Kaplan-Meier curves

The following graph (eFigure 1) depicts the morbidity plotted with the length of hospital stay (oral group: median 21 days, 95% CI 17-25 *vs.* enteral group: median 23 days, 95% CI 17-29, log rank p=0.406).

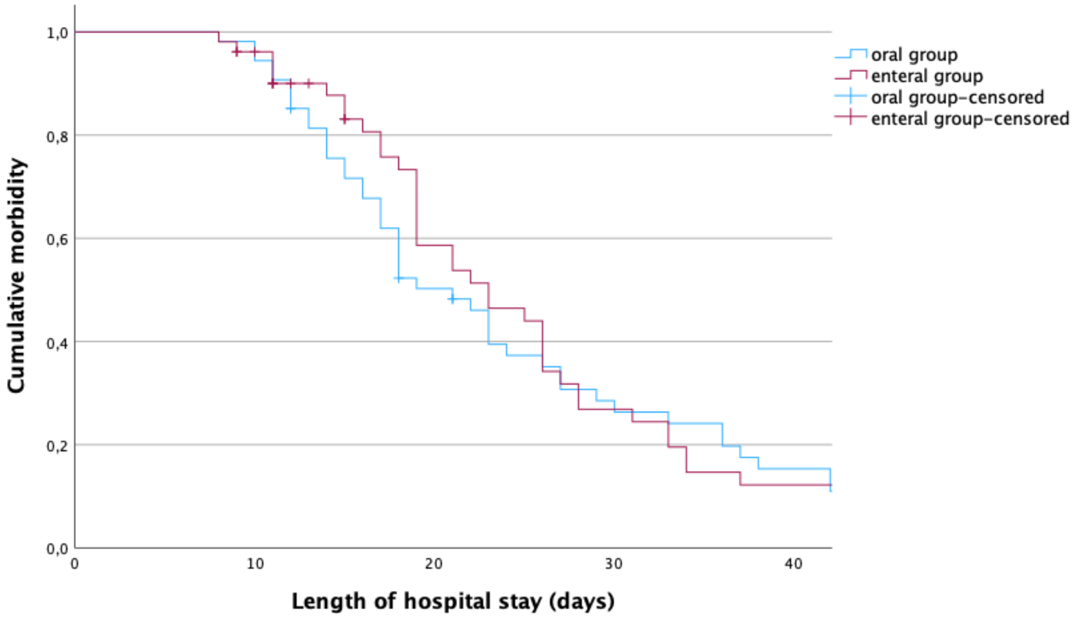

eFigure 1. Kaplan Meier Curve - morbidity plotted with the length of hospital stay

© 2026 Joliat et al. *JAMA Surg*.

Complementary analyses on main outcome

Center-adjusted regression

A center-adjusted linear regression was performed for the CCI (dependent variable). Unstandardized coefficients for the supplemental enteral group and for the centers (A and B/C) were -10.3 (95% CI -18.5 to -2.1, p=0.014) and -16.4 (95% CI -27.3 to -5.4, p=0.004), respectively.

A factorial ANOVA test was performed to account for enteral nutrition, age, diabetes, BMI, biliary drainage, and NRS. Enteral nutrition was found to have an independent effect on the CCI. F statistic for enteral nutrition was 5.1 (p=0.029), and no interactions were significant. The estimated marginal CCI mean was significantly lower for the enteral group (27.4 ± 3.3) compared to the oral group (38.3 ± 3.1, mean difference: -10.9, 95% CI -19.9 to -1.8, p=0.019).

eTable 2. Outcomes stratified by histology and NRS

	Mean CCI, enteral group	Mean CCI oral group	Mean difference (95% CI)	p-value
Malignant etiology	22.7 ± 16.8	37.1 ± 24.2	-14.4 (-23.0 to -5.8)	0.001
Benign etiology	46.5 ± 13.9	47.8 ± 28.4	-1.3 (-31.3 to 28.7)	0.910
NRS 3	28.9 ± 21.8	32.4 ± 23.5	-3.4 (-16.7 to 9.9)	0.607
NRS >3	26.1 ± 14.2	42.2 ± 24.5	-16.0 (-26.7 to -5.4)	0.004

NRS: nutritional risk screening, CCI: comprehensive complication index, CI: confidence interval.

© 2026 Joliat et al. *JAMA Surg*.

References

1. Joliat GR, Labgaa I, Petermann D, et al. Cost-benefit analysis of an enhanced recovery protocol for pancreaticoduodenectomy. *Br J Surg*. 2015;102(13):1676-1683. doi:10.1002/bjs.9957

2. Melloul E, Lassen K, Roulin D, et al. Guidelines for Perioperative Care for Pancreatoduodenectomy: Enhanced Recovery After Surgery (ERAS) Recommendations 2019. *World J Surg*. 2020;44(7):2056-2084. doi:10.1007/s00268-020-05462-w

© 2026 Joliat et al. *JAMA Surg*.
