# Supplementary material for: Early Enteral vs Oral Postoperative Nutrition After Pancreatoduodenectomy: The NUTRIWHI Randomized Clinical Trial
Source: JAMA Surg. 2026 Apr 22;161(6):575–82. doi: 10.1001/jamasurg.2026.1048 (PMC13103875; doi:10.1001/jamasurg.2026.1048)
Supplement: Supplement 3. — Data sharing statement [file jamasurg-e261048-s003.pdf]

Data Sharing Statement

Joliat. Early Enteral vs Oral Postoperative Nutrition After Pancreatoduodenectomy. *JAMA Surg*. Published April 22, 2026. doi:10.1001/jamasurg.2026.1048

Data
Additional Information: ClinicalTrials.gov: NCT05042882. https://clinicaltrials.gov/
Data available: Yes
Data types: Deidentified participant data
How to access data: Request for data should be sent to the corresponding author (gaetan.joliat@gmail.com)
When available: With publication

Supporting Documents
Document types: None

Additional Information
Who can access the data: Data will be available to researchers who provide a methodologically sound proposal.
Types of analyses: Data will be made available for analyses with a specified purpose.
Mechanisms of data availability: Data requestors will need to sign a data access agreement.
